# Supplementary figures and images for: Stabilization of the SARS-CoV-2 Spike Receptor-Binding Domain Using Deep Mutational Scanning and Structure-Based Design
Source: Front Immunol. 2021 Jun 29;12:710263. doi: 10.3389/fimmu.2021.710263 (PMC8276696; doi:10.3389/fimmu.2021.710263)

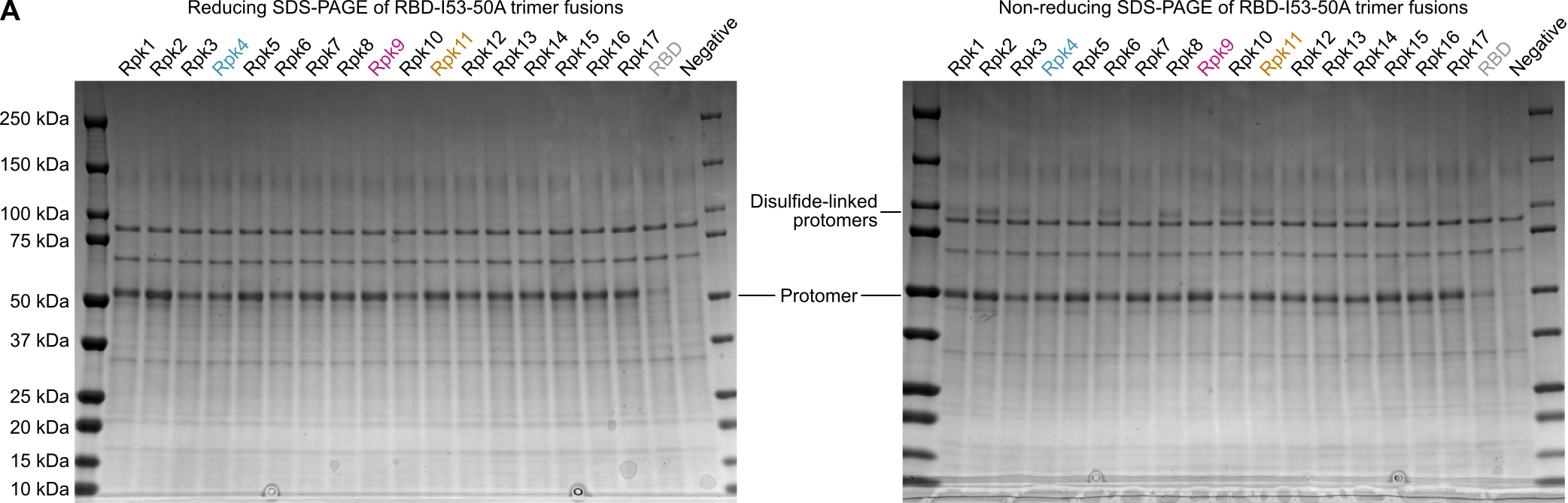

Supplement: Supplementary Figure 1 — Uncropped reducing and non-reducing SDS-PAGE of supernatants from HEK293F cells during expression of stabilized RBD designs genetically fused to the I53-50A trimer. “Negative” refers to a negative control plasmid that does not encode a secreted protein. Cropped gels are shown in Figure 1D . [file Image_1.tiff]

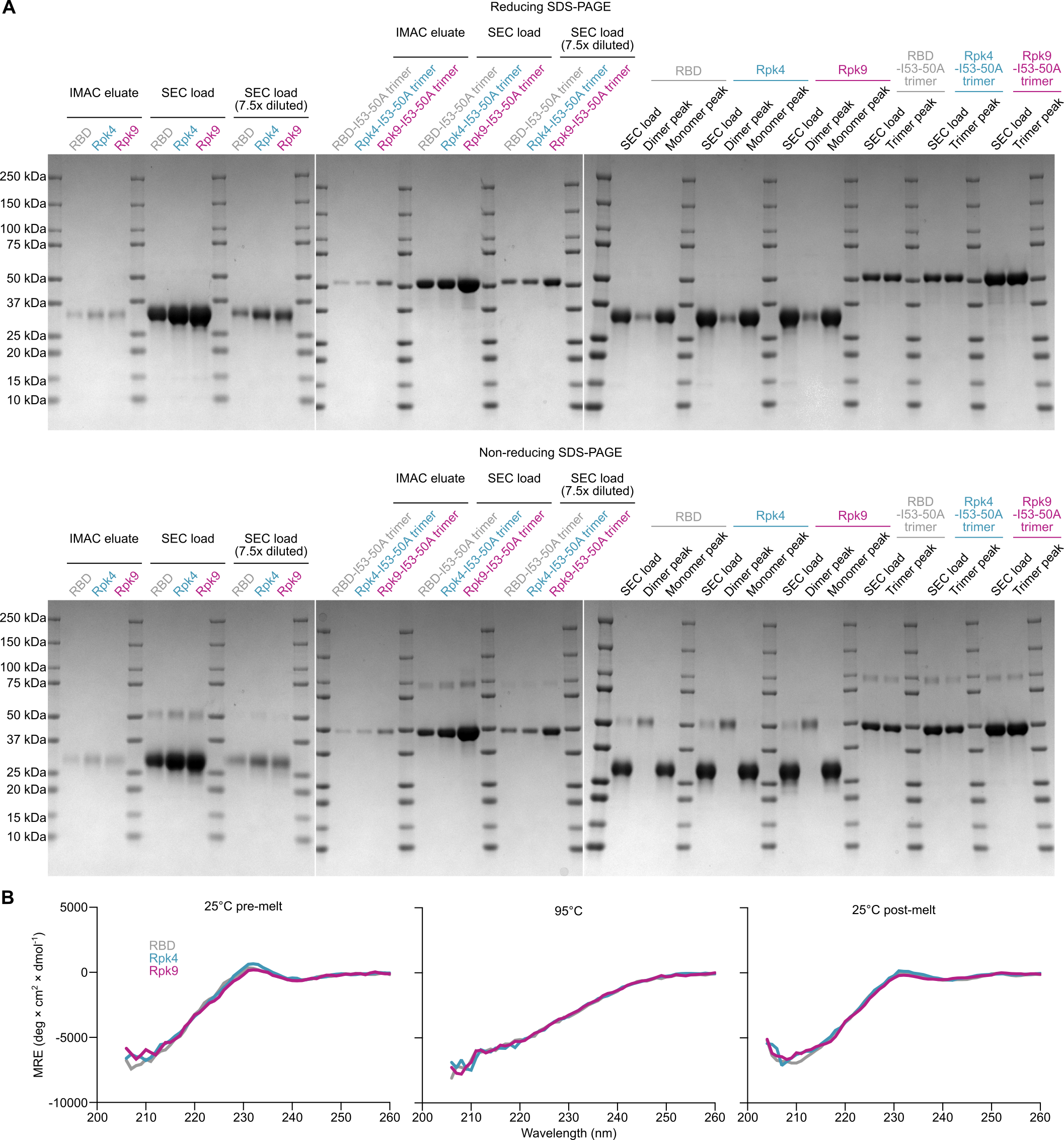

Supplement: Supplementary Figure 2 — SDS-PAGE of purification of RBD monomers and trimers and circular dichroism of RBD monomers. (A) Reducing and non-reducing SDS-PAGE of intermediates and final products during the purification of wild-type and stabilized RBD monomers and genetic fusions to the I53-50A trimer. Selected data are shown cropped in Figure 2A . (B) Circular dichroism spectra of wild-type and stabilized RBD monomers. Spectra were collected initially at 25°C (left), after raising the temperature to 95°C (center), and again after returning the temperature to 25°C (right). [file Image_2.tiff]

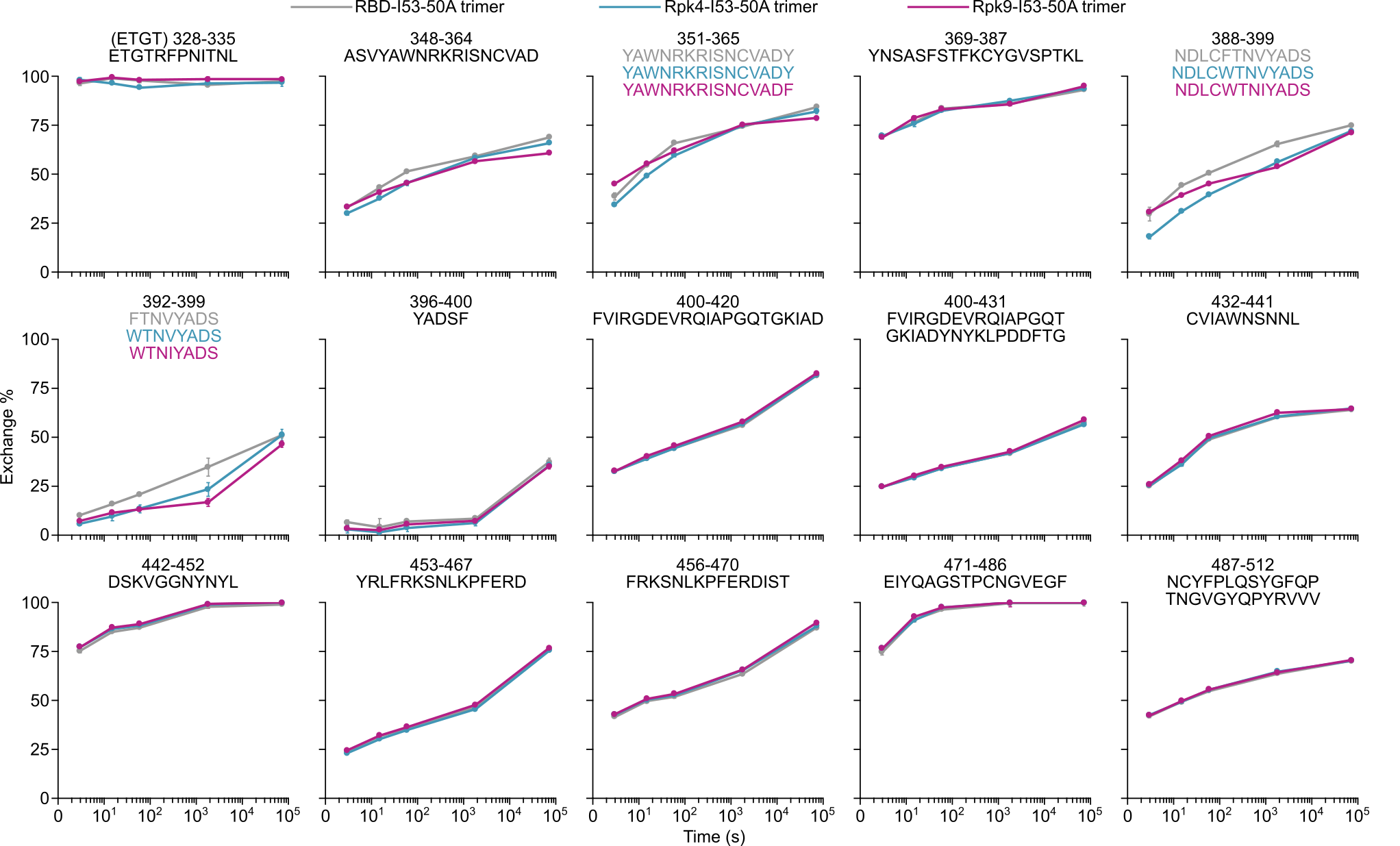

Supplement: Supplementary Figure 3 — HDX-MS of wild-type and stabilized RBD fusions to I53-50A trimers. Kinetics of deuterium uptake for numbered peptides and leading N-terminal segment (top left plot) in all three constructs are shown for 3 sec, 15 sec, 1 min, 30 min, and 20 h timepoints. Each point is an average of two measurements. Standard deviations are shown unless smaller than the points plotted. [file Image_3.tiff]

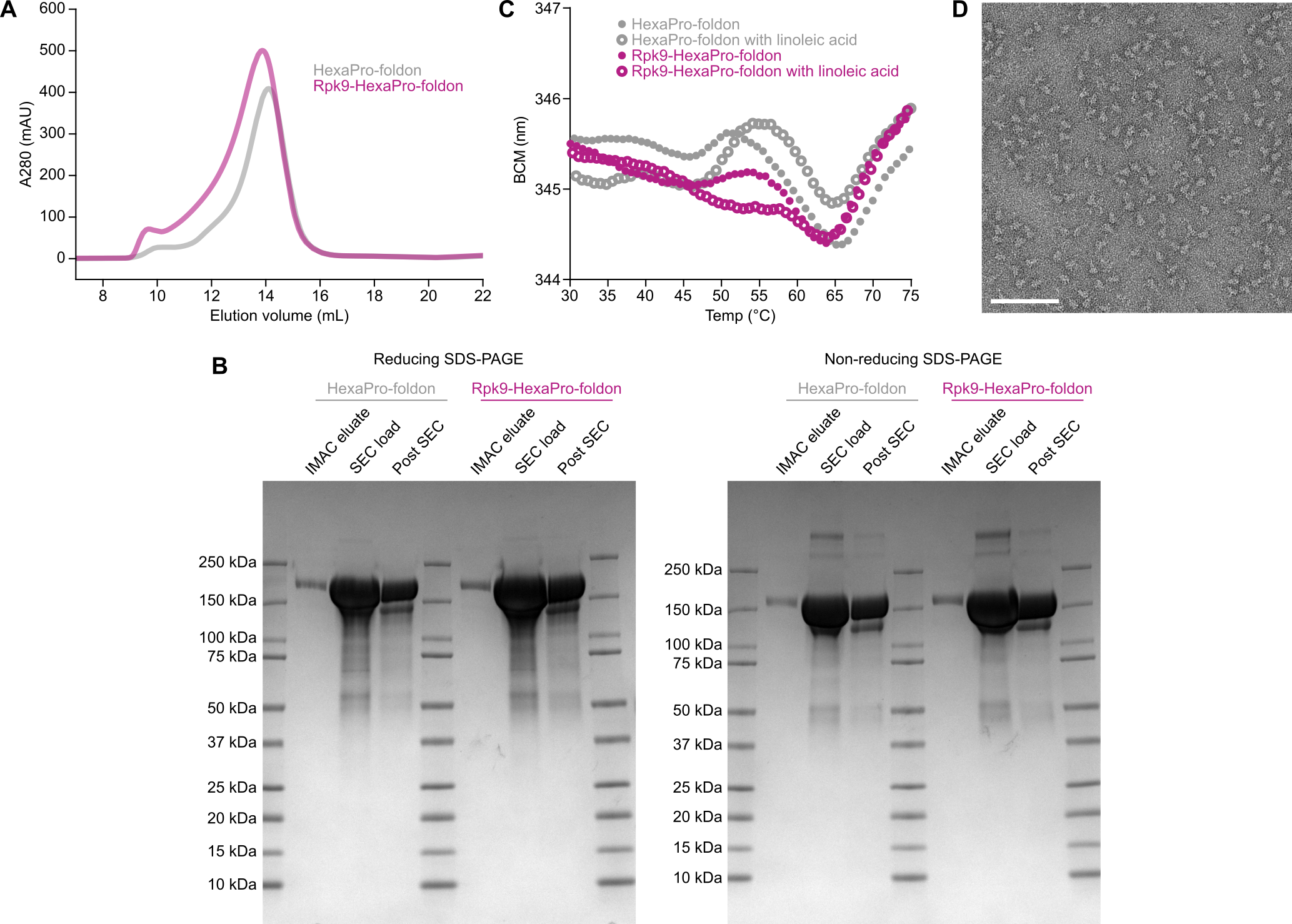

Supplement: Supplementary Figure 4 — Rpk9 mutations can be incorporated into full length SARS-CoV-2 S ectodomains containing HexaPro mutations. (A) SEC purification of wild-type (HexaPro-foldon) and Rpk9 (Rpk9-HexaPro-foldon) prefusion-stabilized S ectodomains after expression from equal volumes of HEK293F cultures followed by IMAC purification and concentration. S ectodomains were purified using a Superose 6 Increase 10/300 GL. (B) Reducing and non-reducing SDS-PAGE of intermediates and final products during the purification of HexaPro-foldon and Rpk9-HexaPro-foldon. (C) Thermal denaturation of HexaPro-foldon and Rpk9-HexaPro-foldon either in the presence of a 40-fold molar excess (290 μM) of linoleic acid (open circles) or not (closed circles), monitored by nanoDSF using intrinsic tryptophan fluorescence. The barycentric mean (BCM) of the fluorescence emission spectra is plotted as a function of temperature. (D) nsEM of Rpk9-HexaPro-foldon (scale bar, 100 nm). [file Image_4.tiff]

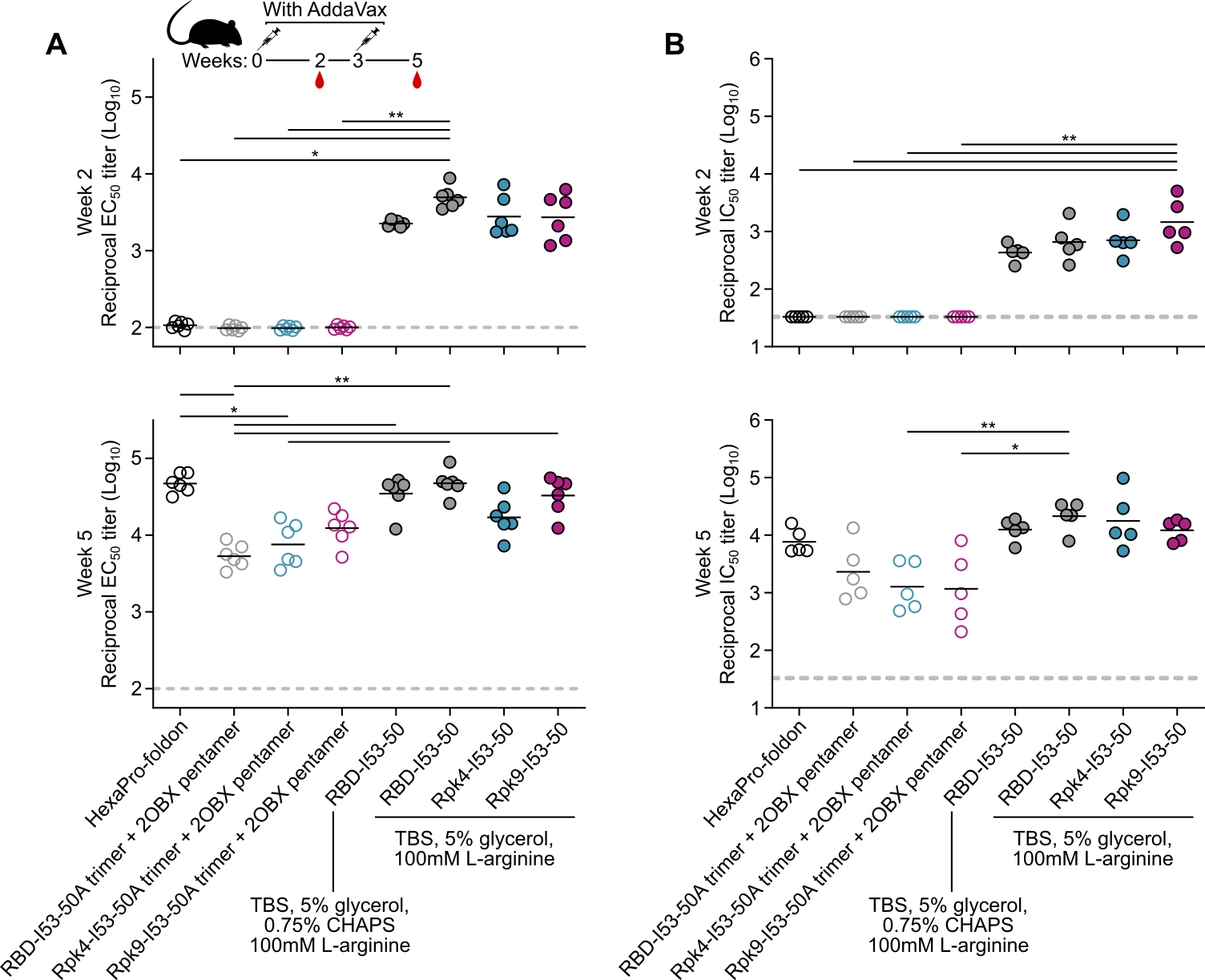

Supplement: Supplementary Figure 5 — Midpoint binding titers and MLV-based pseudovirus neutralizing titers elicited by RBD immunogens in mice. (A) Serum binding against HexaPro-foldon at weeks 2 and 5 as assessed by midpoint titers (EC50) from ELISA measurements. Each circle represents the titer at 50% binding for an individual mouse, and horizontal lines show the geometric mean of each group. (B) Autologous pseudovirus neutralization using an MLV backbone. Each circle represents the neutralizing antibody titer at 50% inhibition (IC50) for an individual mouse and horizontal lines show the geometric mean of each group. Five randomly selected mice were analyzed from each group. Statistical analysis was performed using one-sided nonparametric Kruskal–Wallis test with Dunn’s multiple comparisons. *p < 0.05; **p < 0.01. [file Image_5.tiff]
