# Supplementary material for: Stabilization of the SARS-CoV-2 Spike Receptor-Binding Domain Using Deep Mutational Scanning and Structure-Based Design
Source: Front Immunol. 2021 Jun 29;12:710263. doi: 10.3389/fimmu.2021.710263 (PMC8276696; doi:10.3389/fimmu.2021.710263)
Supplement: Supplementary file 6 [file Table_1.docx]

**Supplementary Table 1** | Mutations included in each stabilized RBD design. Mutations are separated into previously reported DMS-identified mutations [(57)](https://paperpile.com/c/glHrWD/iSYUv) or mutations identified by Rosetta.

| **Mutation set** | **Mutations identified by DMS** | **Mutations identified by Rosetta** |
| --- | --- | --- |
| Rpk1 | Y365W |  |
| Rpk2 | Y365W | F338L |
| Rpk3 | Y365W | L513M |
| Rpk4 | F392W |  |
| Rpk5 | Y365W, F392W |  |
| Rpk6 | Y365F | F338M, A363L, F377V |
| Rpk7 | Y365F, F392W |  |
| Rpk8 | Y365F | V395I |
| Rpk9 | Y365F, F392W | V395I |
| Rpk10 | Y365W | L513I, F515L |
| Rpk11 |  | F338L, A363L, Y365M |
| Rpk12 | I358F, Y365W |  |
| Rpk13 | I358F, Y365W | F338L |
| Rpk14 | I358F, Y365W | L513M |
| Rpk15 | I358F, Y365F | V395I |
| Rpk16 | I358F, Y365W, F392W |  |
| Rpk17 | I358F, Y365F, F392W | V395I |
