# Supplementary material for: Stabilization of the SARS-CoV-2 Spike Receptor-Binding Domain Using Deep Mutational Scanning and Structure-Based Design
Source: Front Immunol. 2021 Jun 29;12:710263. doi: 10.3389/fimmu.2021.710263 (PMC8276696; doi:10.3389/fimmu.2021.710263)
Supplement: Supplementary file 9 [file DataSheet_3.pdf]

Supplementary Information 3

Supplementary Solution Stability Data

SDS-PAGE for nanoparticles in TBS, 5% glycerol, 0.75% CHAPS, 100 mM L-arginine

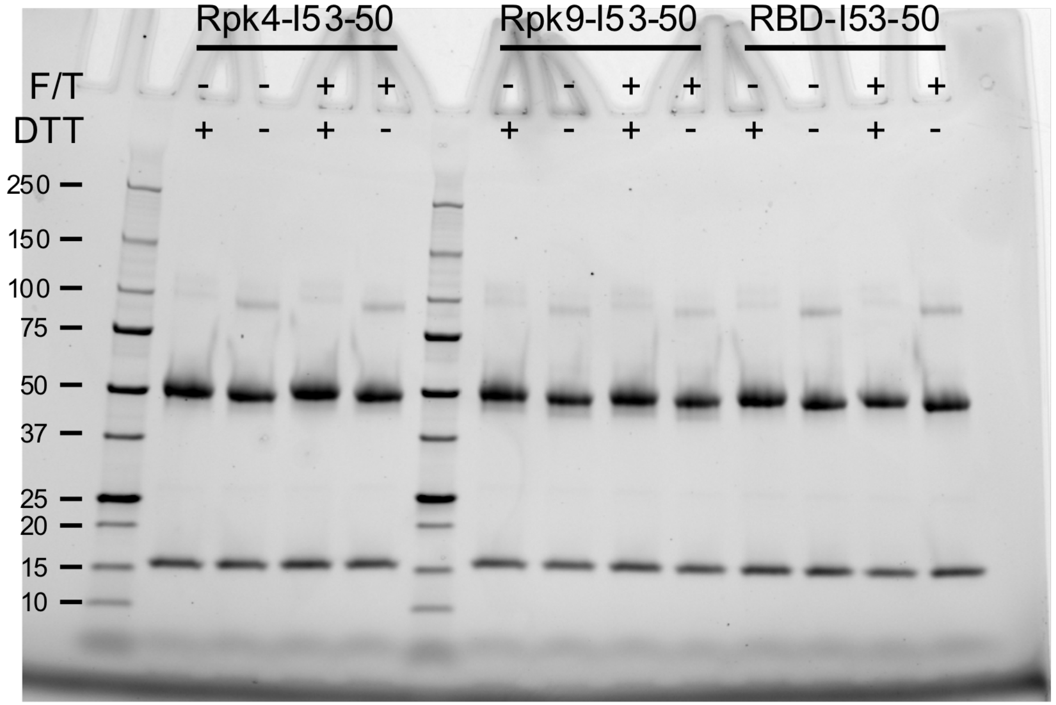

The integrity of samples in 50 mM Tris pH 7.4, 185 mM NaCl, 4.5% glycerol, 0.75% CHAPS, 100 mM L-arginine was analyzed by SDS-PAGE. Molecular weights of the standard are noted in kDa. Each sample was analyzed +/- reducing agent (DTT), pre- and post-freeze thaw (F/T).

hACE2-Fc binding for nanoparticles in TBS, 5% glycerol, 0.75% CHAPS, 100 mM L-arginine

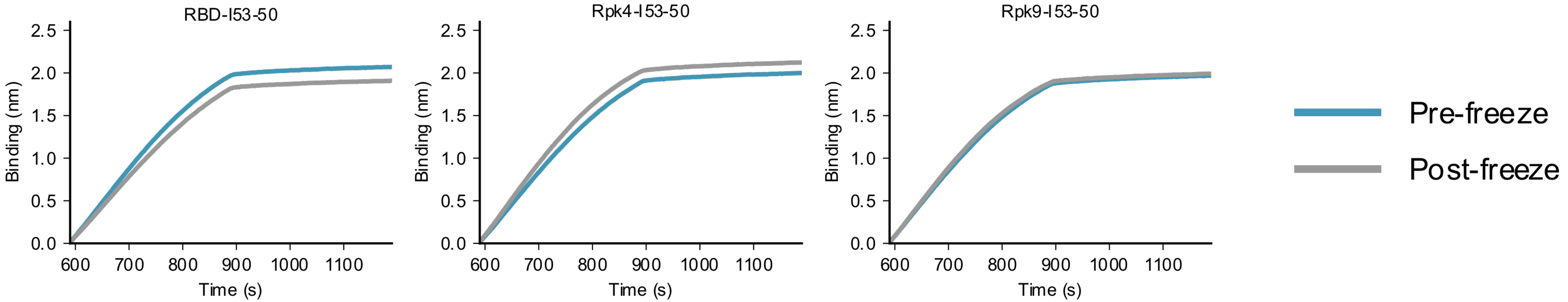

hACE2-Fc binding of antigen in 50 mM Tris pH 7.4, 185 mM NaCl, 4.5% glycerol, 0.75% CHAPS, 100 mM L-arginine was analyzed by bio-layer interferometry (BLI). Protein A biosensors loaded with hACE2-Fc were incubated with immunogen (association, x = 590–889 s) and then buffer (dissociation, x = 890–1190 s).

CR3022 binding for nanoparticles in TBS, 5% glycerol, 0.75% CHAPS, 100 mM L-arginine

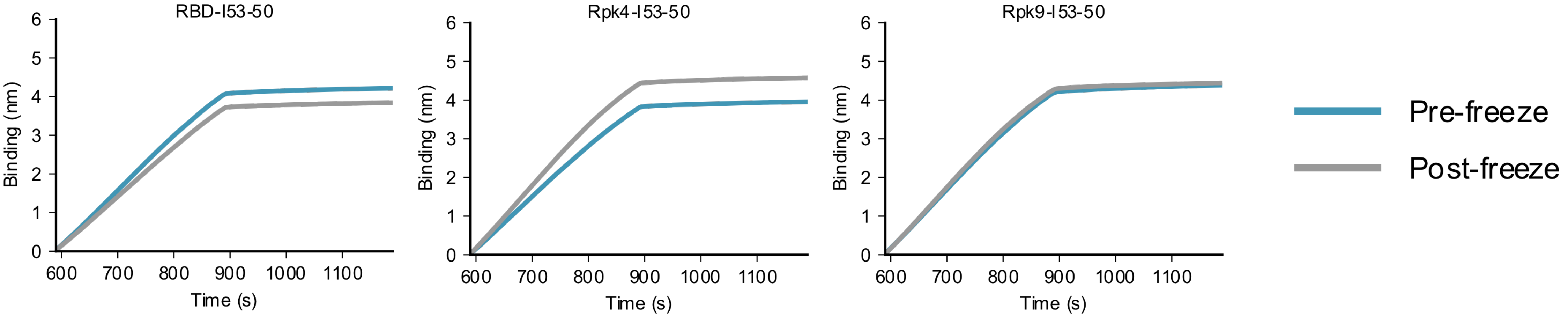

CR3022 IgG binding of antigen in 50 mM Tris pH 7.4, 185 mM NaCl, 4.5% glycerol, 0.75% CHAPS, 100 mM L-arginine was analyzed by bio-layer interferometry (BLI). Protein A biosensors loaded with CR3022 IgG were incubated with immunogen (association, x = 590–889 s) and then buffer (dissociation, x = 890–1190 s).

Dynamic light scattering for nanoparticles in TBS, 5% glycerol, 0.75% CHAPS, 100 mM L-arginine

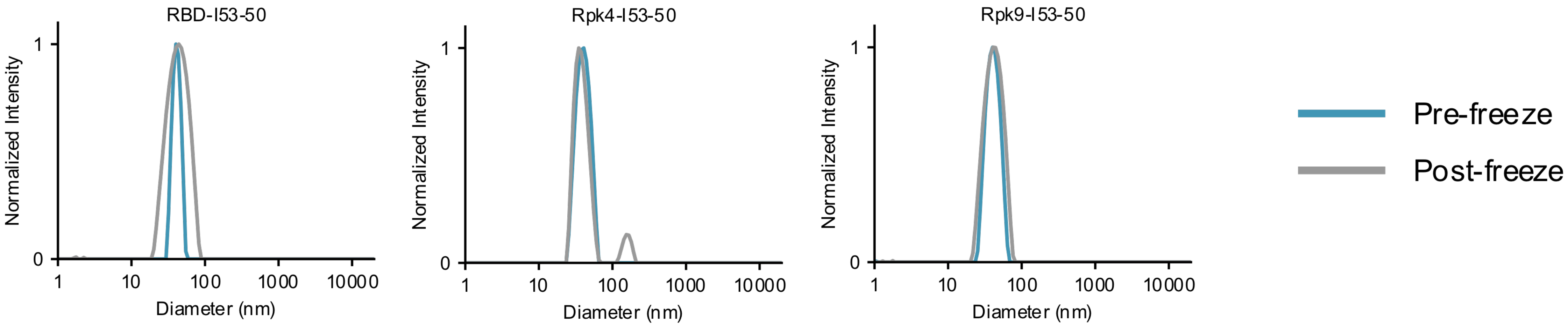

Hydrodynamic diameter (nm) for each sample in 50 mM Tris pH 7.4, 185 mM NaCl, 4.5% glycerol, 0.75% CHAPS, 100 mM L-arginine, plotted as normalized intensity.

UV-Vis for nanoparticles in TBS, 5% glycerol, 0.75% CHAPS, 100 mM L-arginine

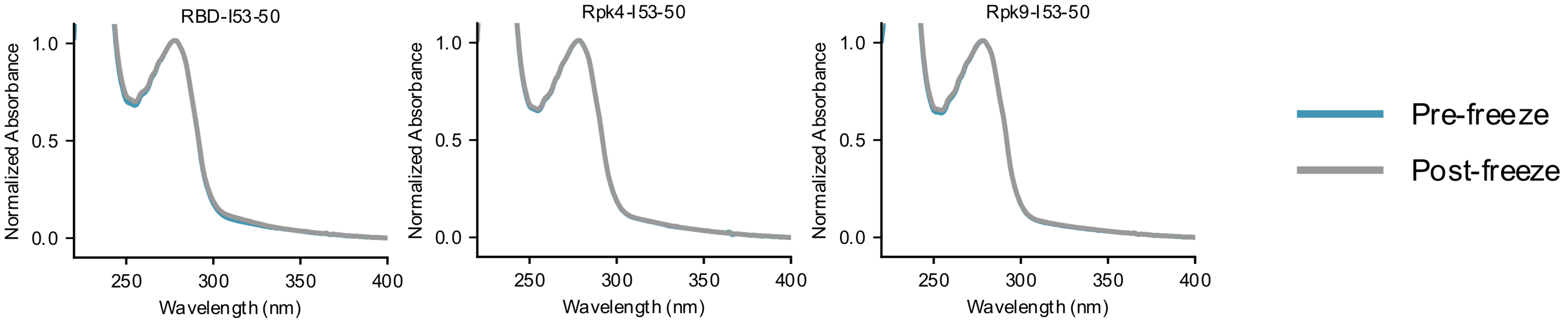

UV-Vis spectra (nm) for each sample in 50 mM Tris pH 7.4, 185 mM NaCl, 4.5% glycerol, 0.75% CHAPS, 100 mM L-arginine, plotted as normalized absorbance.

SDS-PAGE for nanoparticles in TBS, 5% glycerol, 100 mM L-arginine

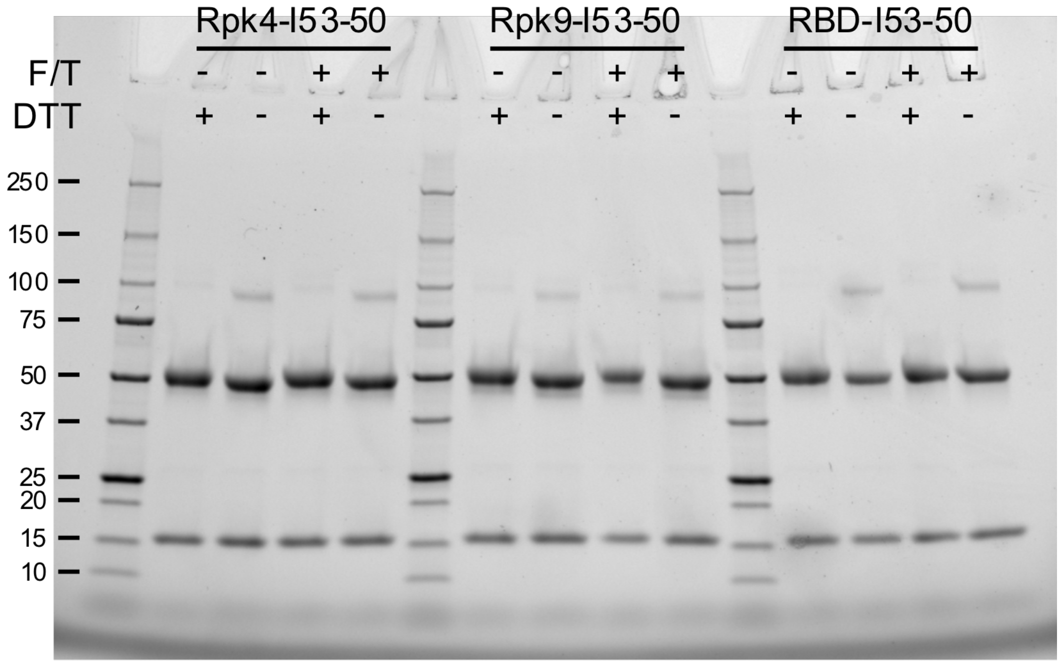

The integrity of samples in 50 mM Tris pH 8, 150 mM NaCl, 5% glycerol, 100 mM L-arginine was analyzed by SDS-PAGE. Molecular weights of the standard are noted in kDa. Each sample was analyzed +/- reducing agent (DTT), pre- and post-freeze thaw (F/T).

hACE2-Fc binding for nanoparticles in TBS, 5% glycerol, 100 mM L-arginine

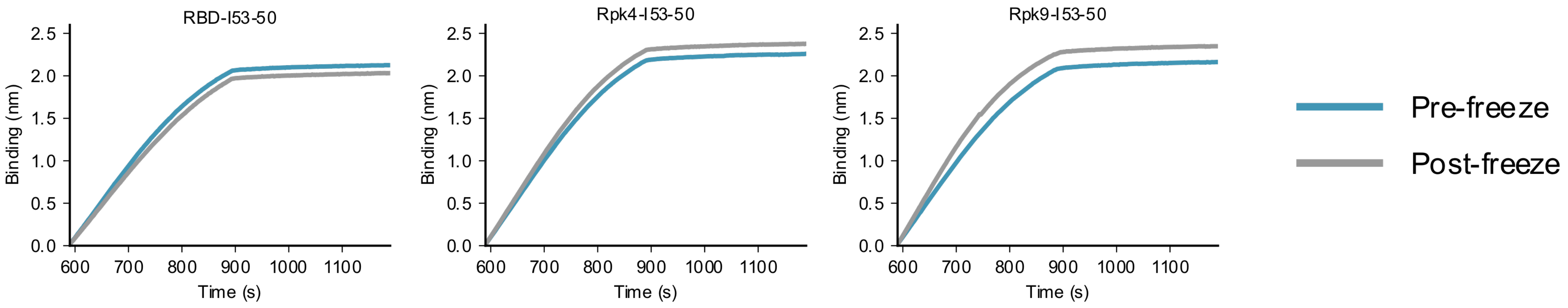

hACE2-Fc binding of antigen in 50 mM Tris pH 8, 150 mM NaCl, 5% glycerol, 100 mM L-arginine was analyzed by bio-layer interferometry (BLI). Protein A biosensors loaded with hACE2-Fc were incubated with immunogen (association, x = 590–889 s) and then buffer (dissociation, x = 890–1190 s).

CR3022 binding for nanoparticles in TBS, 5% glycerol, 100 mM L-arginine

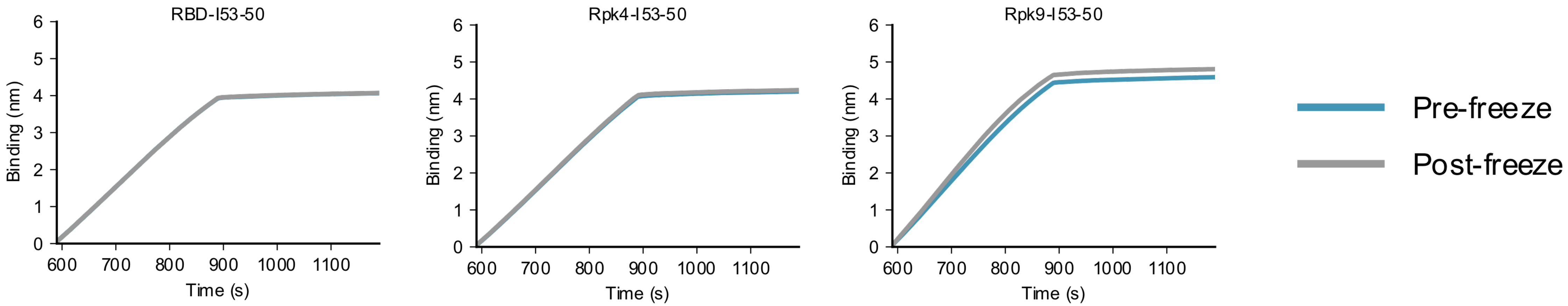

CR3022 IgG binding of antigen in 50 mM Tris pH 8, 150 mM NaCl, 5% glycerol, 100 mM L-arginine was analyzed by bio-layer interferometry (BLI). Protein A biosensors loaded with CR3022 IgG were incubated with immunogen (association, x = 590–889 s) and then buffer (dissociation, x = 890–1190 s).

Dynamic light scattering for nanoparticles in TBS, 5% glycerol, 100 mM L-arginine

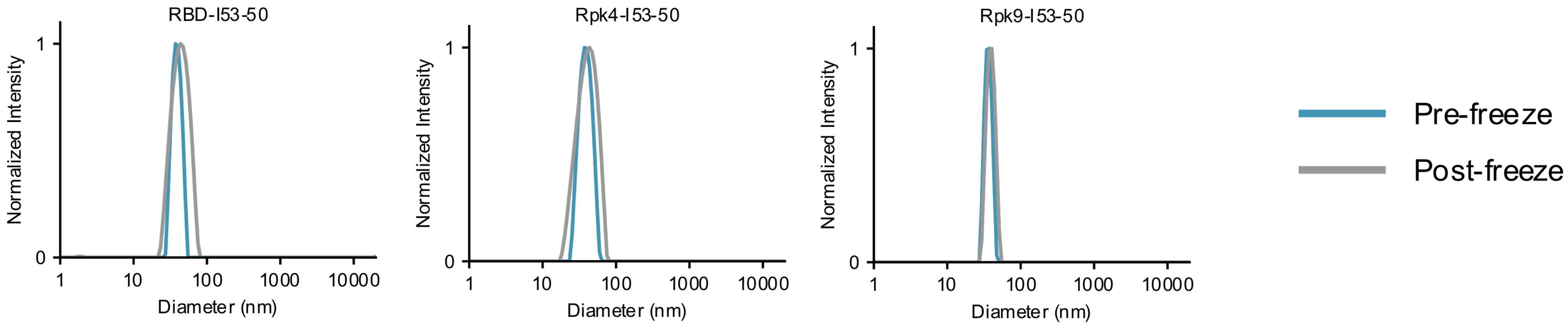

Hydrodynamic diameter (nm) for each sample in 50 mM Tris pH 8, 150 mM NaCl, 5% glycerol, 100 mM L-arginine, plotted as normalized intensity.

UV-Vis for nanoparticles in TBS, 5% glycerol, 100 mM L-arginine

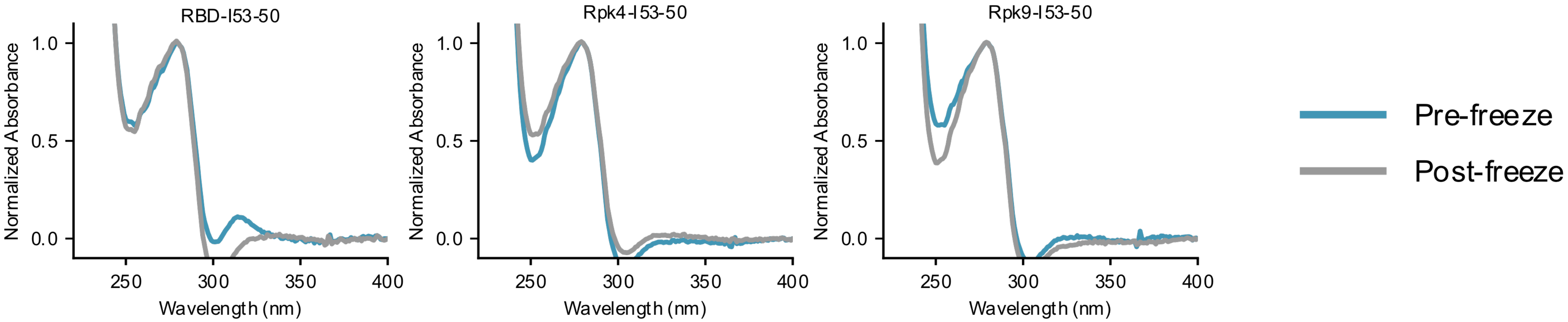

UV-Vis spectra (nm) for each sample in 50 mM Tris pH 8, 150 mM NaCl, 5% glycerol, 100 mM L-arginine, plotted as normalized absorbance.

SDS-PAGE for nanoparticles in TBS, 5% glycerol

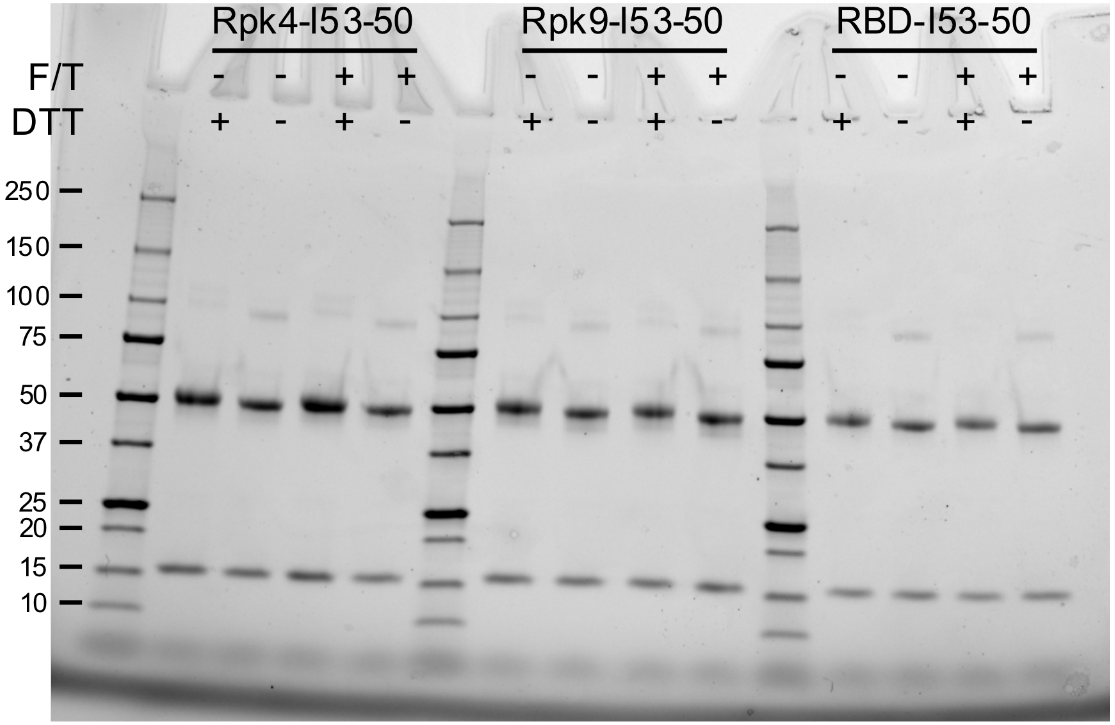

The integrity of samples in 50 mM Tris pH 8, 150 mM NaCl, 5% glycerol was analyzed by SDS-PAGE. Molecular weights of the standard are noted in kDa. Each sample was analyzed +/- reducing agent (DTT), pre- and post-freeze thaw (F/T).

hACE2-Fc binding for nanoparticles in TBS, 5% glycerol

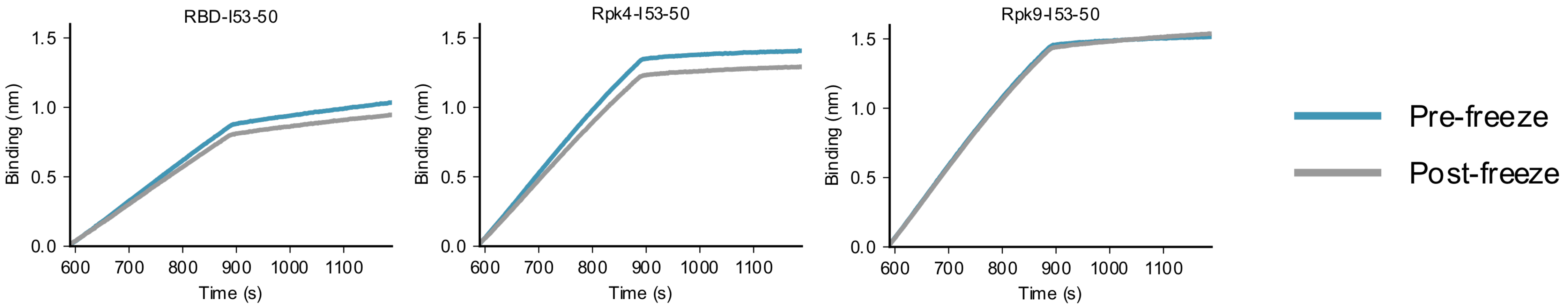

hACE2-Fc binding of antigen in 50 mM Tris pH 8, 150 mM NaCl, 5% glycerol was analyzed by bio-layer interferometry (BLI). Protein A biosensors loaded with hACE2-Fc were incubated with immunogen (association, x = 590–889 s) and then buffer (dissociation, x = 890–1190 s).

CR3022 binding for nanoparticles in TBS, 5% glycerol

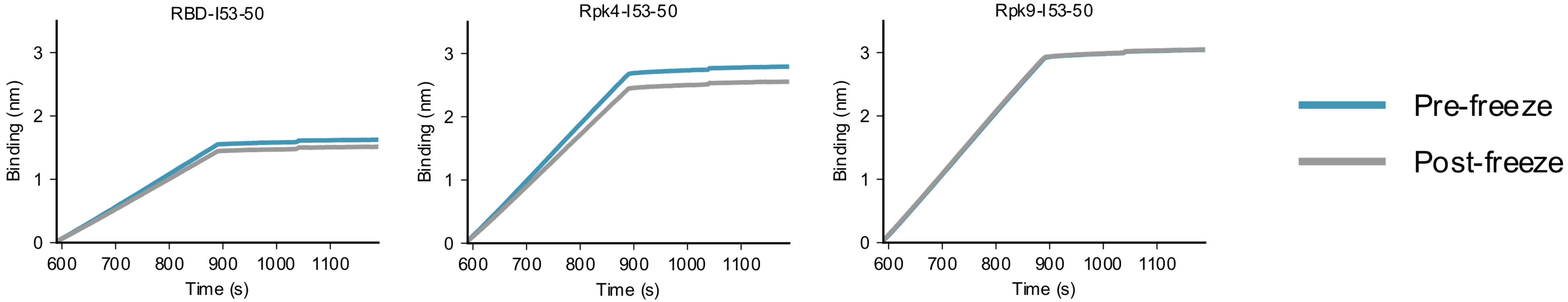

CR3022 IgG binding of antigen in 50 mM Tris pH 8, 150 mM NaCl, 5% glycerol was analyzed by bio-layer interferometry (BLI). Protein A biosensors loaded with CR3022 IgG were incubated with immunogen (association, x = 590–889 s) and then buffer (dissociation, x = 890–1190 s).

Dynamic light scattering for nanoparticles in TBS, 5% glycerol

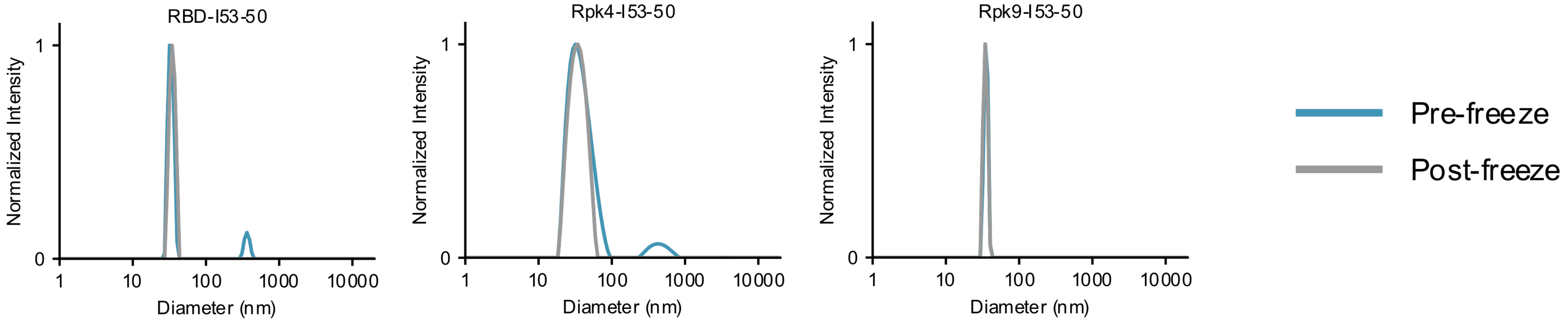

Hydrodynamic diameter (nm) for each sample in 50 mM Tris pH 8, 150 mM NaCl, 5% glycerol, plotted as normalized intensity.

UV-Vis for nanoparticles in TBS, 5% glycerol

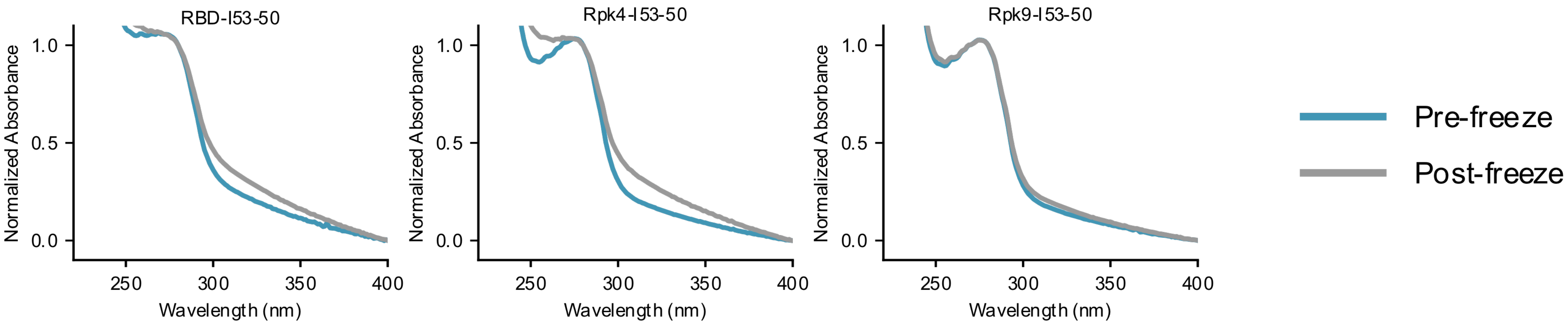

UV-Vis spectra (nm) for each sample in 50 mM Tris pH 8, 150 mM NaCl, 5% glycerol, plotted as normalized absorbance.

SDS-PAGE for nanoparticles in TBS

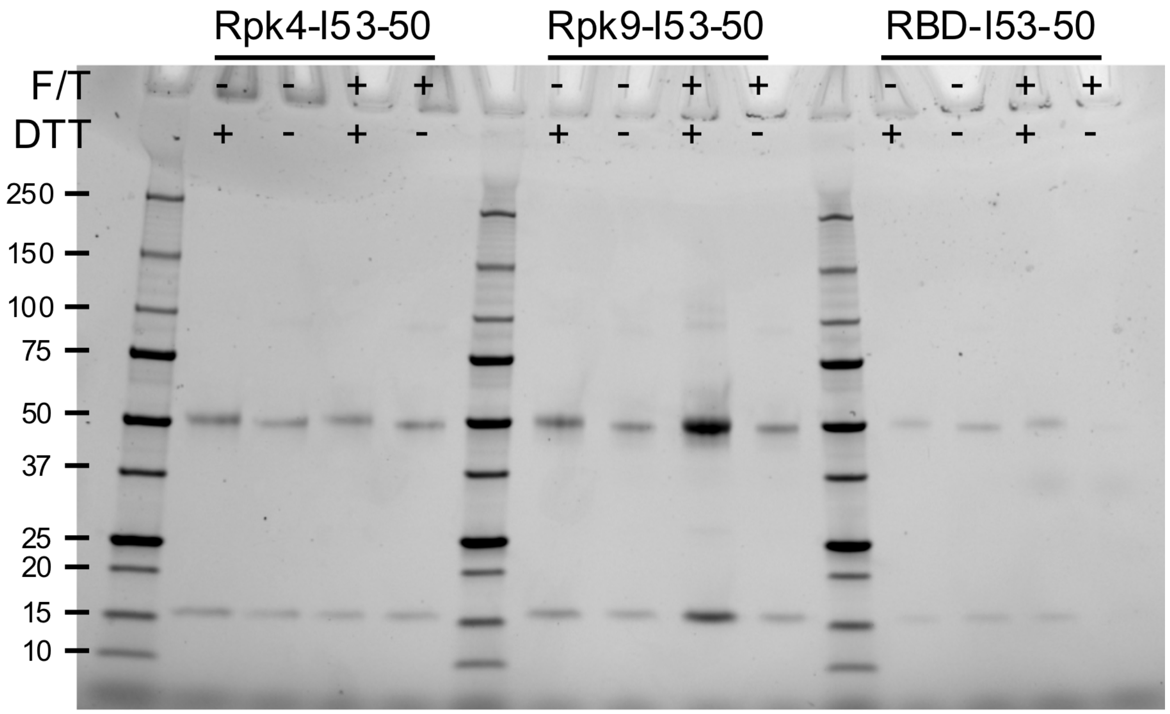

The integrity of samples in 50 mM Tris pH 8, 150 mM NaCl was analyzed by SDS-PAGE. Molecular weights of the standard are noted in kDa. Each sample was analyzed +/- reducing agent (DTT), pre- and post-freeze thaw (F/T).

hACE2-Fc binding for nanoparticles in TBS

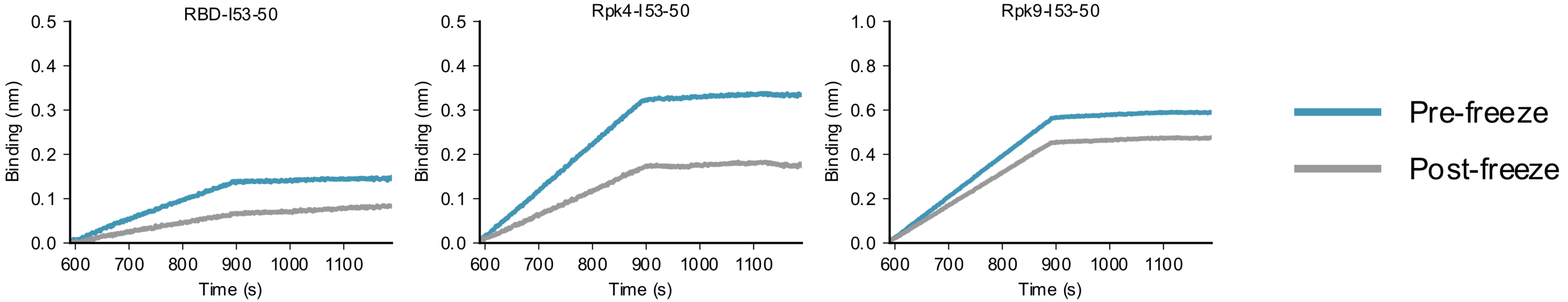

hACE2-Fc binding of antigen in 50 mM Tris pH 8, 150 mM NaCl was analyzed by bio-layer interferometry (BLI). Protein A biosensors loaded with hACE2-Fc were incubated with immunogen (association, x = 590–889 s) and then buffer (dissociation, x = 890–1190 s).

CR3022 binding for nanoparticles in TBS

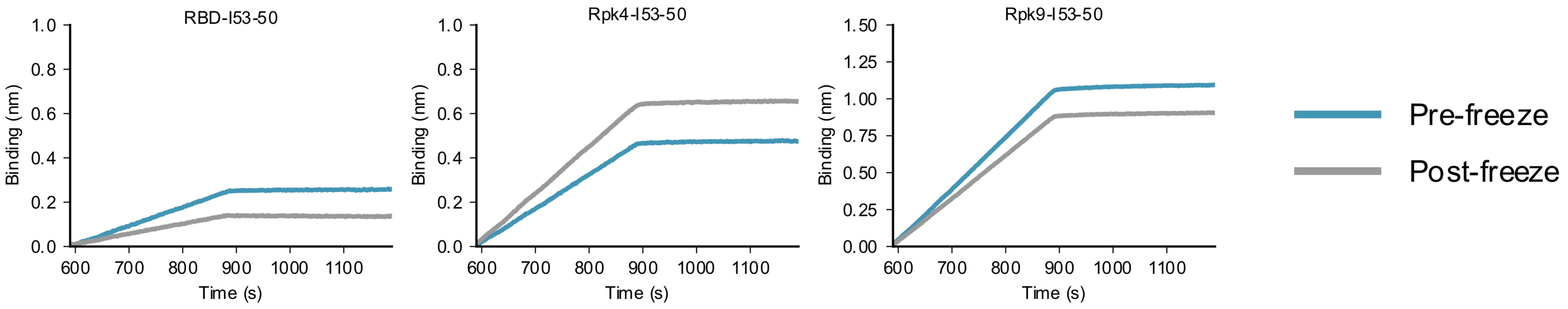

CR3022 IgG binding of antigen in 50 mM Tris pH 8, 150 mM NaCl was analyzed by bio-layer interferometry (BLI). Protein A biosensors loaded with CR3022 IgG were incubated with immunogen (association, x = 590–889 s) and then buffer (dissociation, x = 890–1190 s).

Dynamic light scattering for nanoparticles in TBS

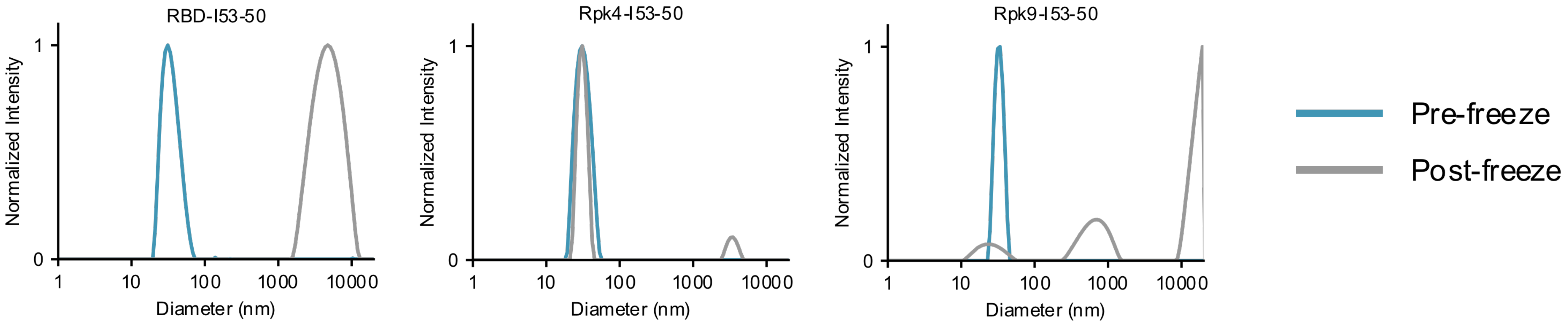

Hydrodynamic diameter (nm) for each sample in 50 mM Tris pH 8, 150 mM NaCl, plotted as normalized intensity.

UV-Vis for nanoparticles in TBS

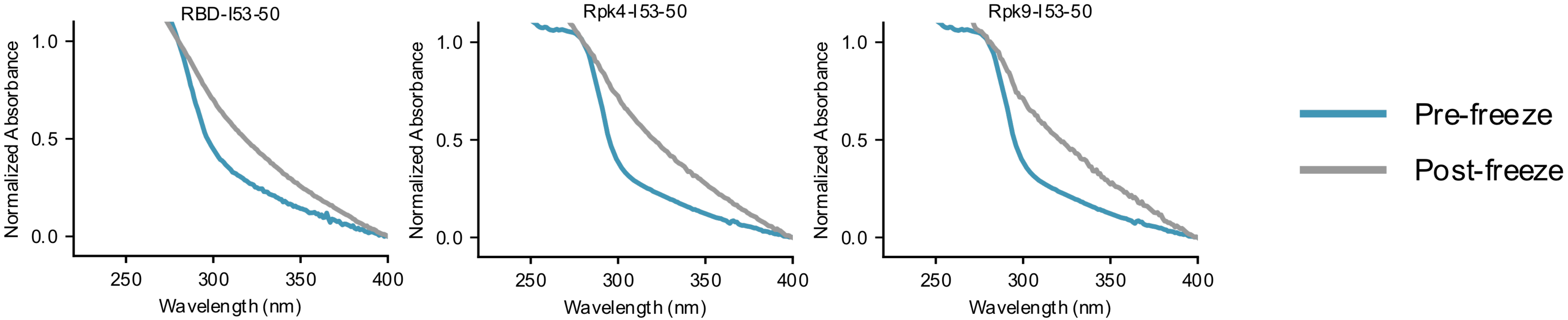

UV-vis spectra (nm) for each sample in 50 mM Tris pH 8, 150 mM NaCl, plotted as normalized absorbance.
