# Supplementary material for: Stabilization of the SARS-CoV-2 Spike Receptor-Binding Domain Using Deep Mutational Scanning and Structure-Based Design
Source: Front Immunol. 2021 Jun 29;12:710263. doi: 10.3389/fimmu.2021.710263 (PMC8276696; doi:10.3389/fimmu.2021.710263)
Supplement: Supplementary file 10 [file DataSheet_4.pdf]

Supplementary Information 4

Supplementary Shelf-life Stability Data

SDS-PAGE for RBD-I53-50 nanoparticle

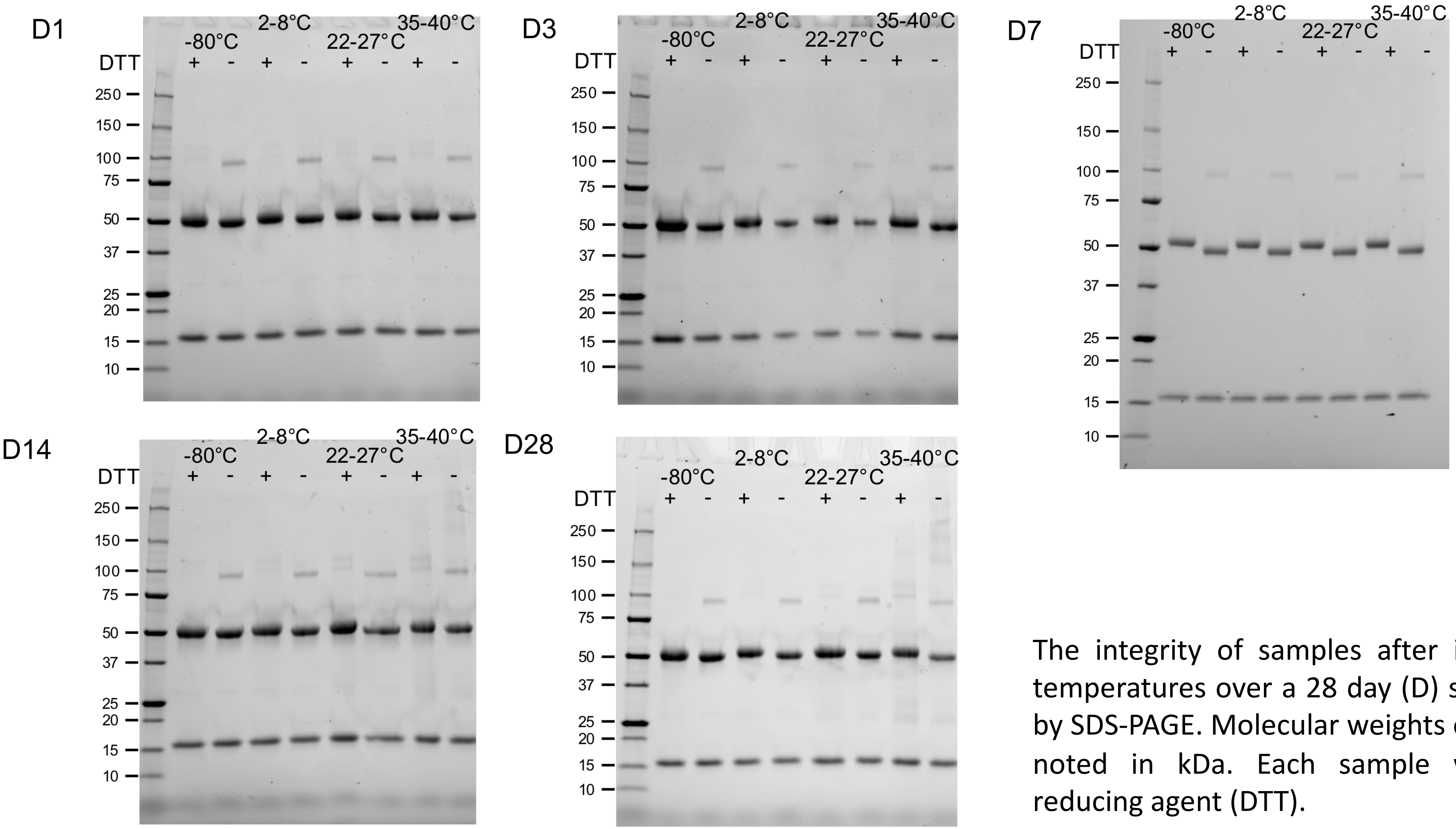

The integrity of samples after incubation at four temperatures over a 28 day (D) study was analyzed by SDS-PAGE. Molecular weights of the standard are noted in kDa. Each sample was analyzed +/- reducing agent (DTT).

hACE2-Fc binding for RBD-I53-50 nanoparticle

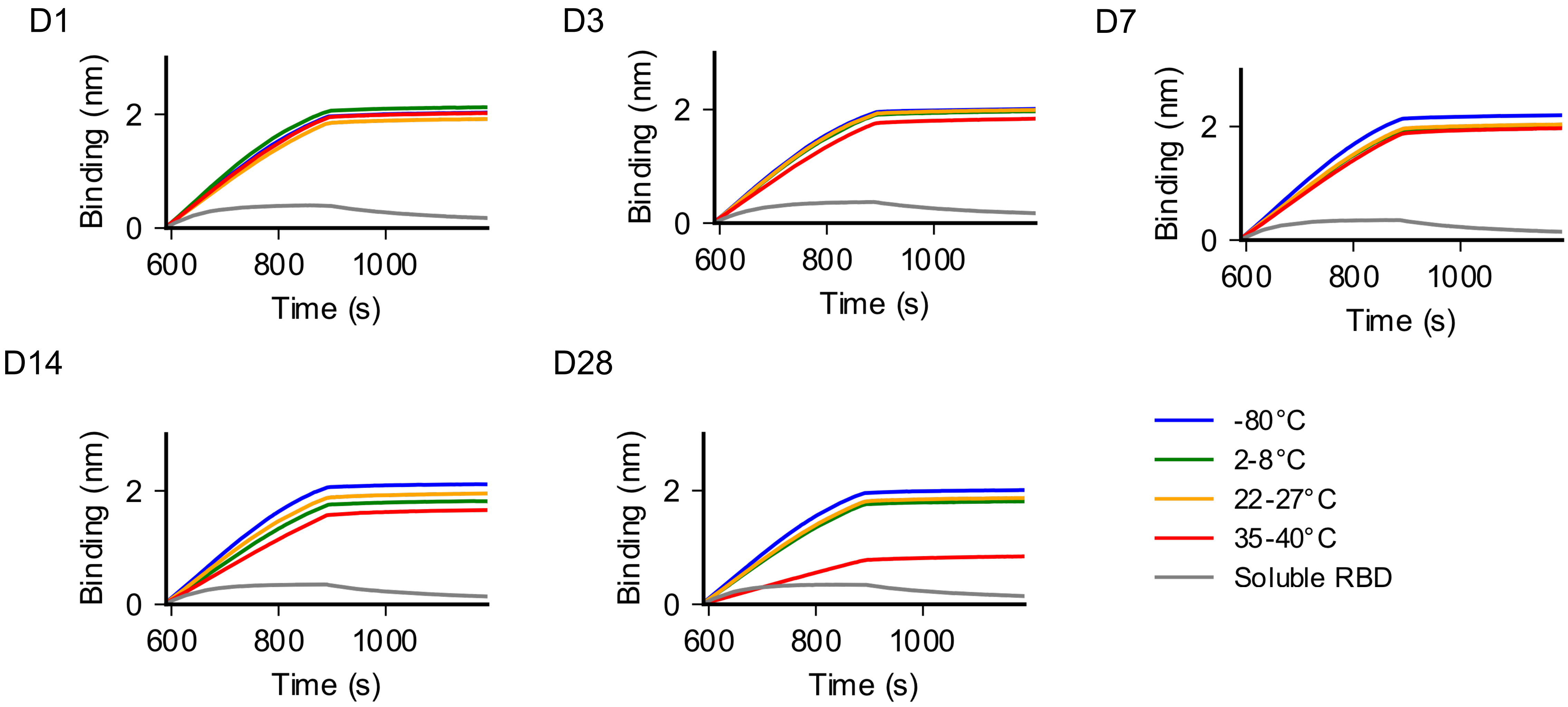

hACE2-Fc binding of antigen incubated at four different temperatures for 28 days (D) was analyzed by bio-layer Interferometry (BLI). Protein A biosensors loaded with hACE2-Fc were incubated with immunogen (association, x = 590–889 s) and then buffer (dissociation, x = 890–1190 s).

CR3022 binding for RBD-I53-50 nanoparticle

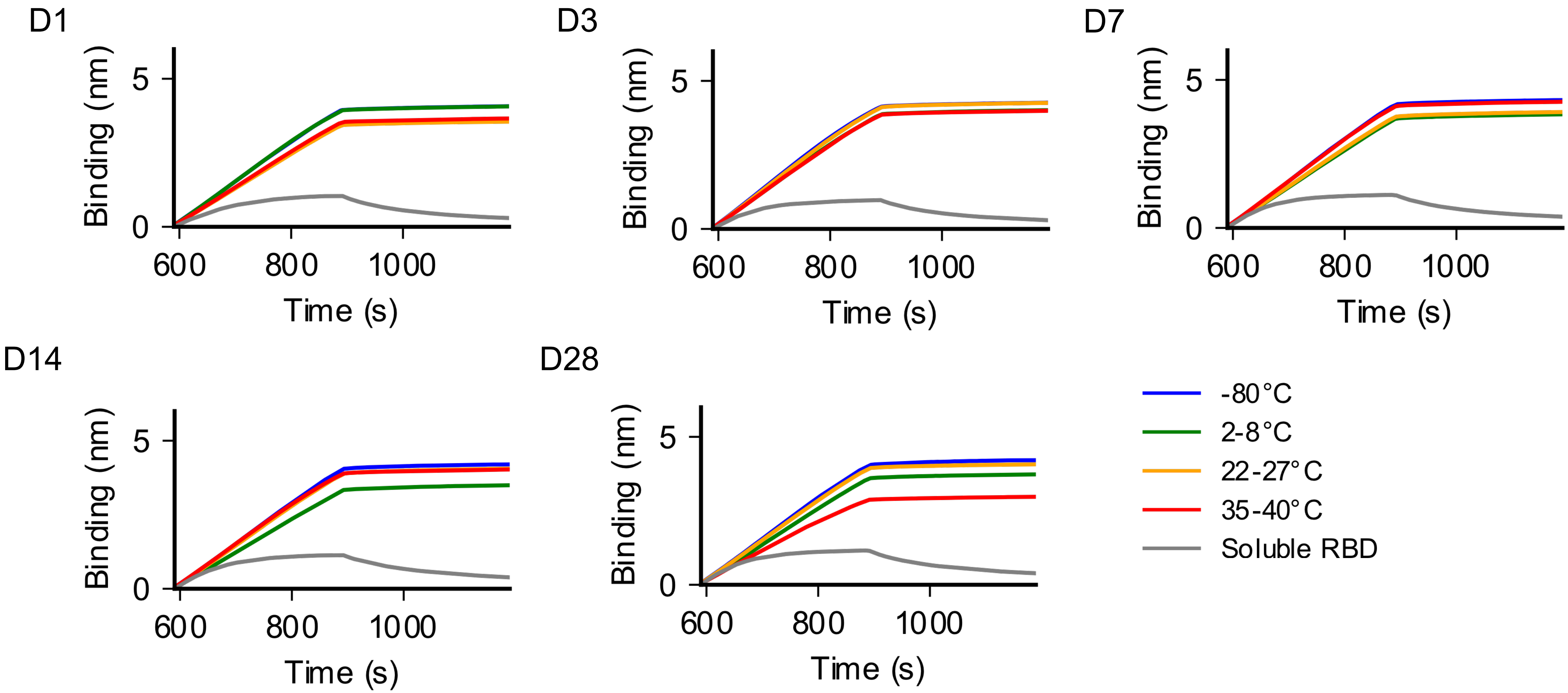

CR3022 IgG binding of antigen incubated at four different temperatures for 28 days (D) was analyzed by bio-layer Interferometry (BLI). Protein A biosensors loaded with CR3022 were incubated with immunogen (association, x = 590–889 s) and then buffer (dissociation, x = 890–1190 s).

nsEM for RBD-I53-50 nanoparticle

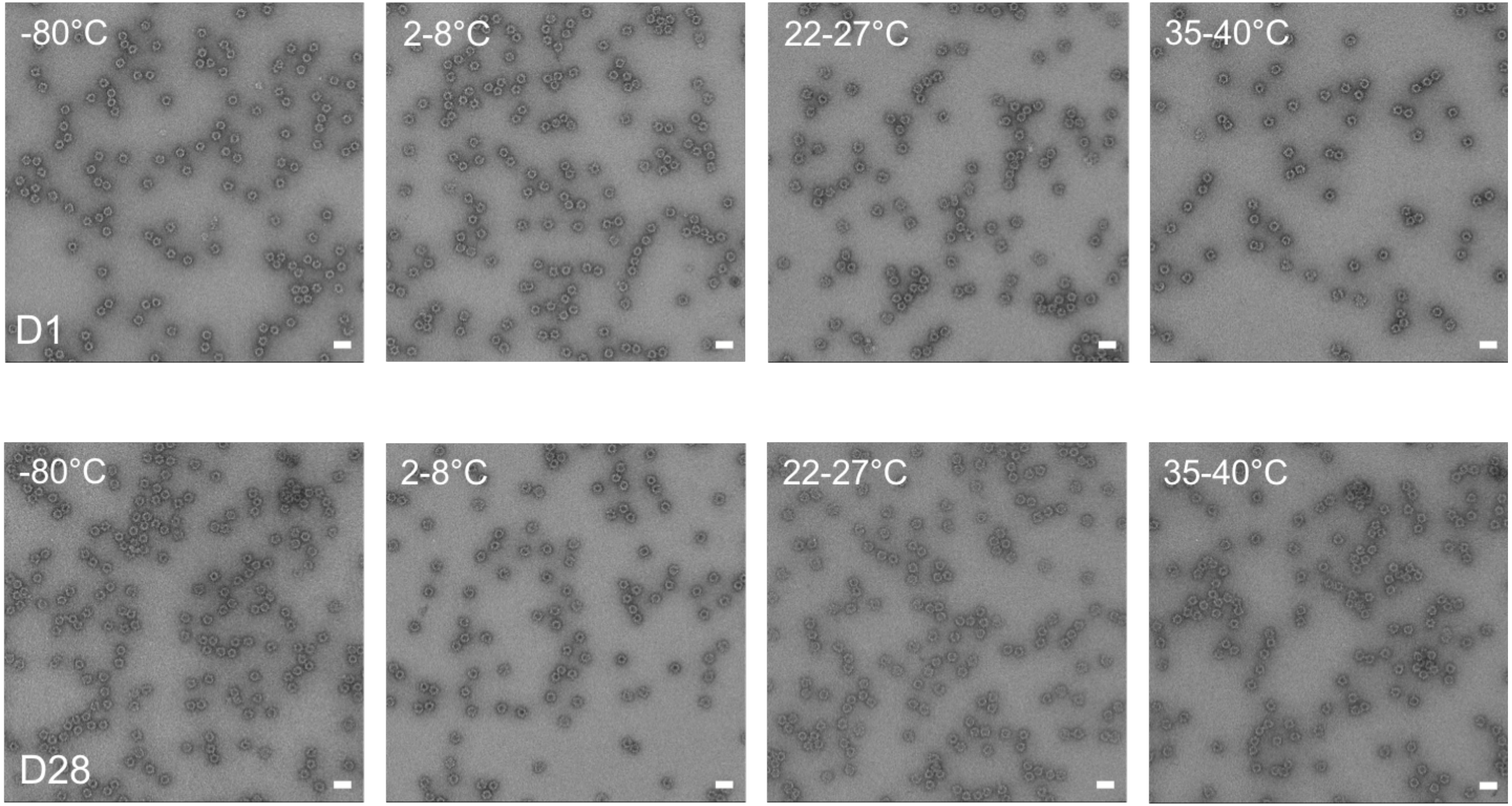

Representative negative stain electron micrographs for each sample at days (D) 1 and 28 following incubation at four temperatures. Scale bar, 50 nm.

Dynamic light scattering for RBD-I53-50 nanoparticle

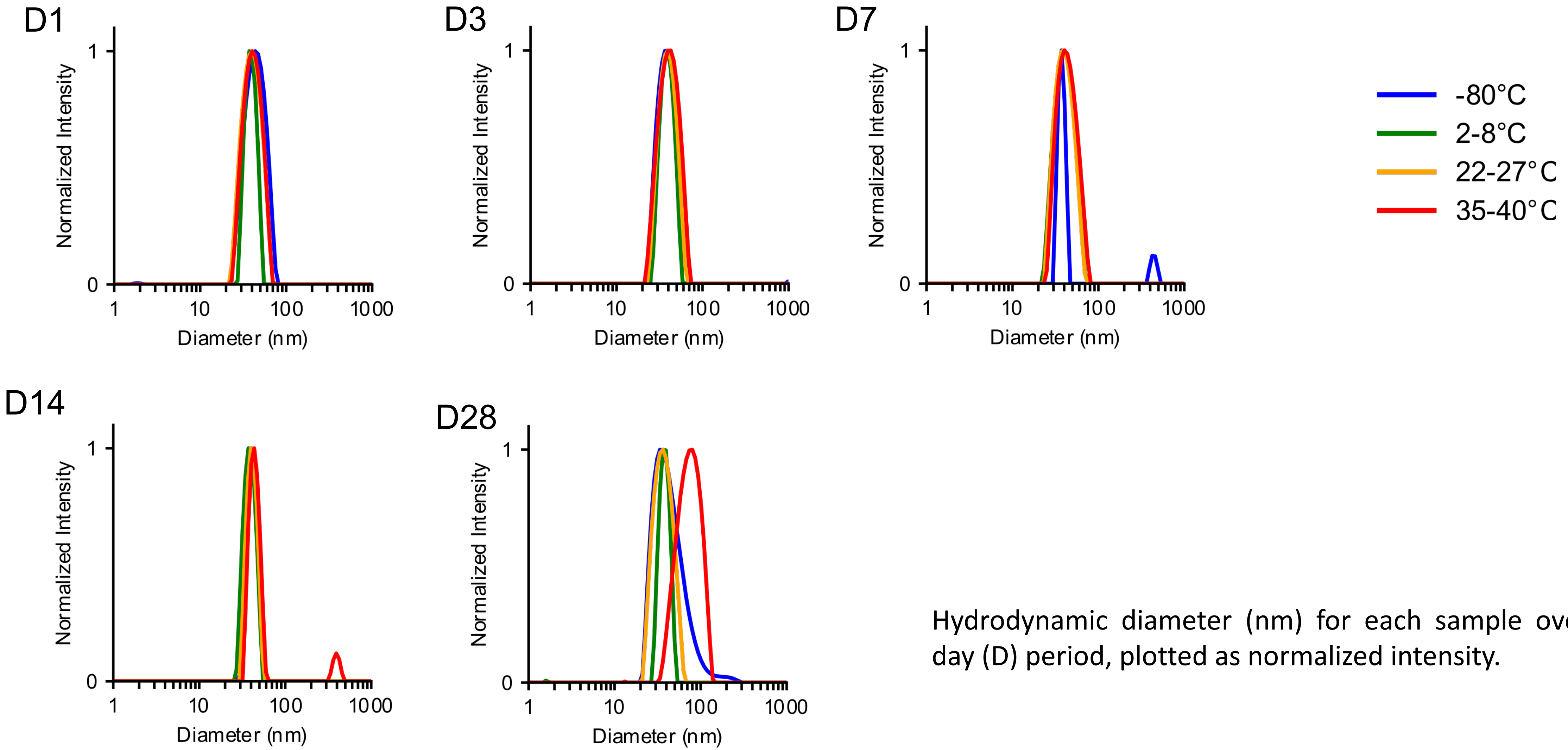

Hydrodynamic diameter (nm) for each sample over a 28 day (D) period, plotted as normalized intensity.

SDS-PAGE for Rpk4-I53-50 nanoparticle

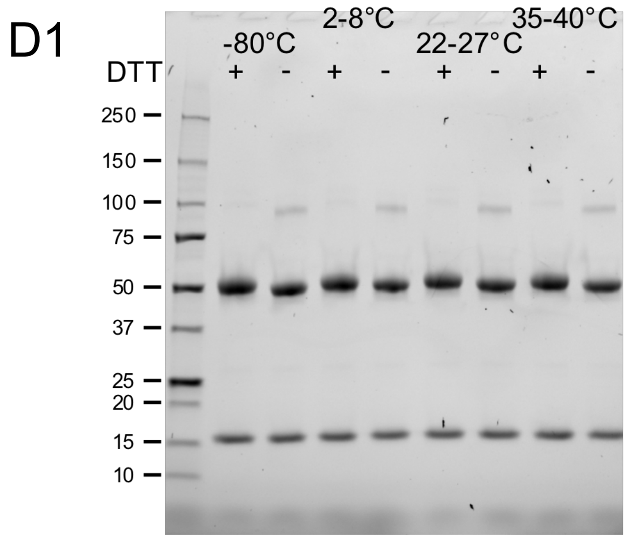

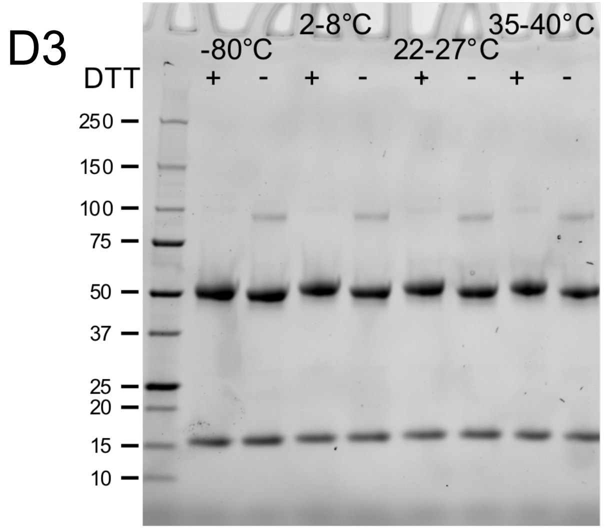

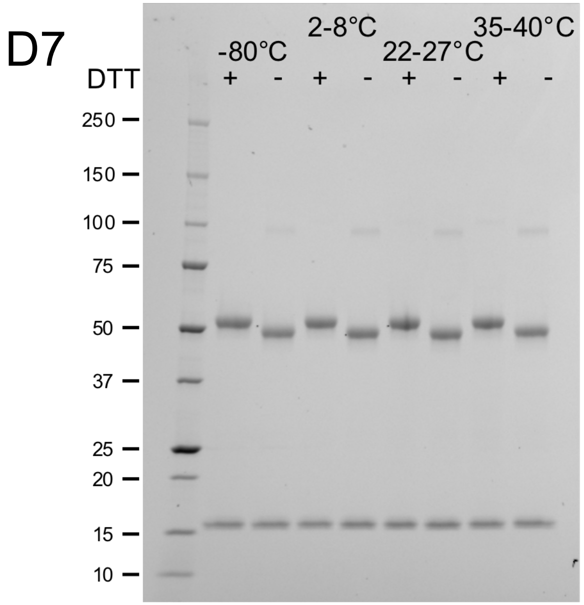

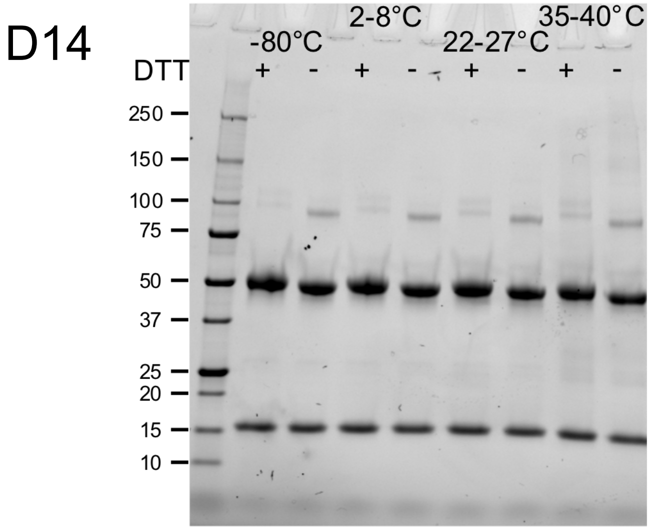

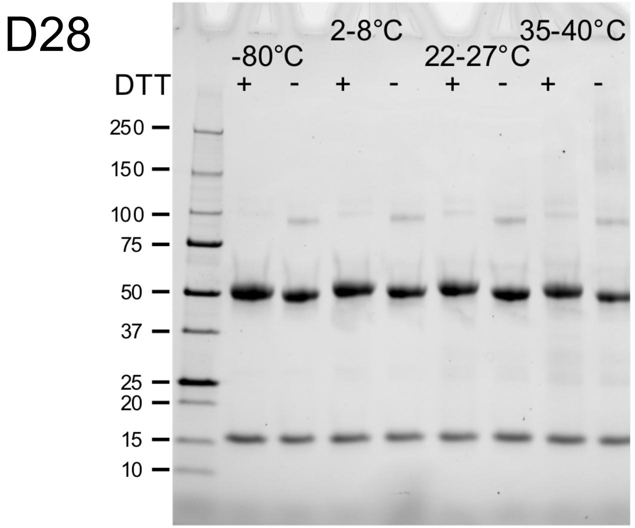

The integrity of samples after incubation at four temperatures over a 28 day (D) study was analyzed by SDS-PAGE. Molecular weights of the standard are noted in kDa. Each sample was analyzed +/- reducing agent (DTT).

hACE2-Fc binding for Rpk4-I53-50 nanoparticle

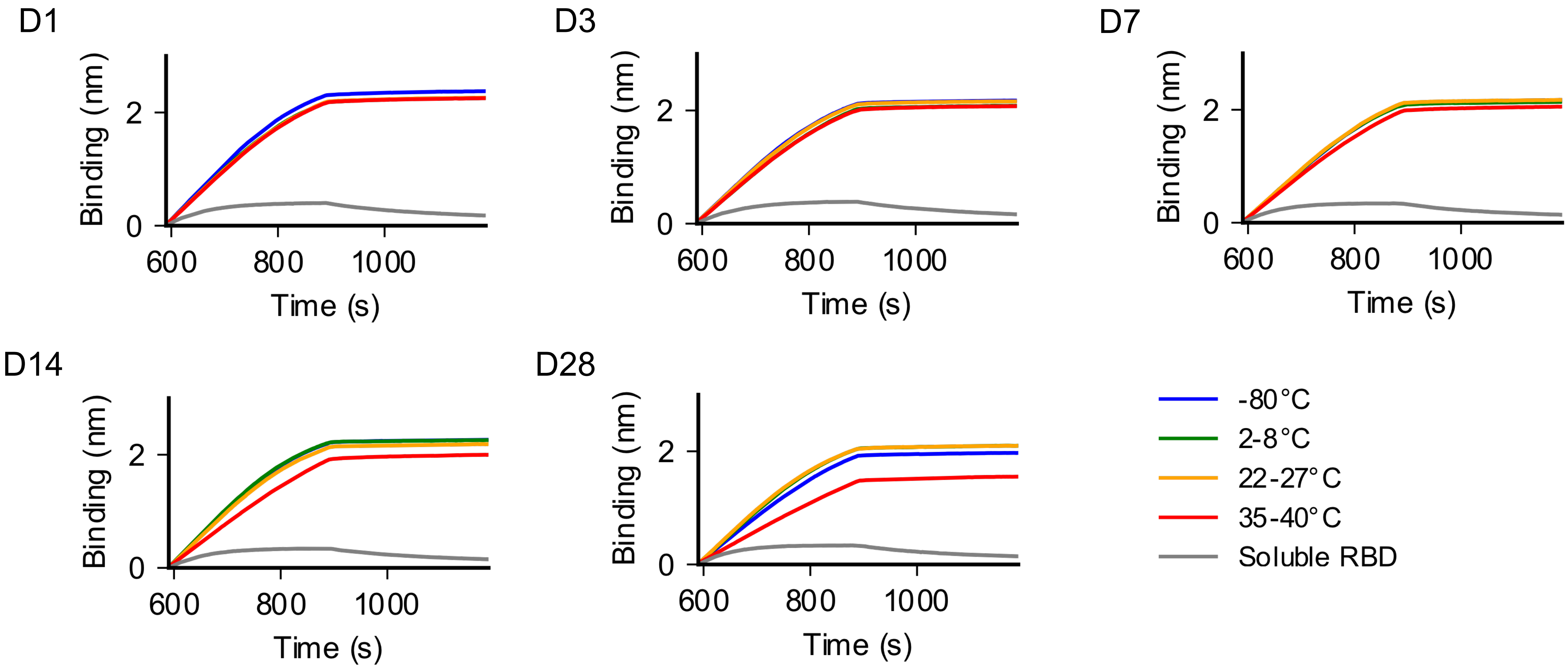

hACE2-Fc binding of antigen incubated at four different temperatures for 28 days (D) was analyzed by bio-layer Interferometry (BLI). Protein A biosensors loaded with hACE2-Fc were incubated with immunogen (association, x = 590–889 s) and then buffer (dissociation, x = 890–1190 s).

CR3022 binding for Rpk4-I53-50 nanoparticle

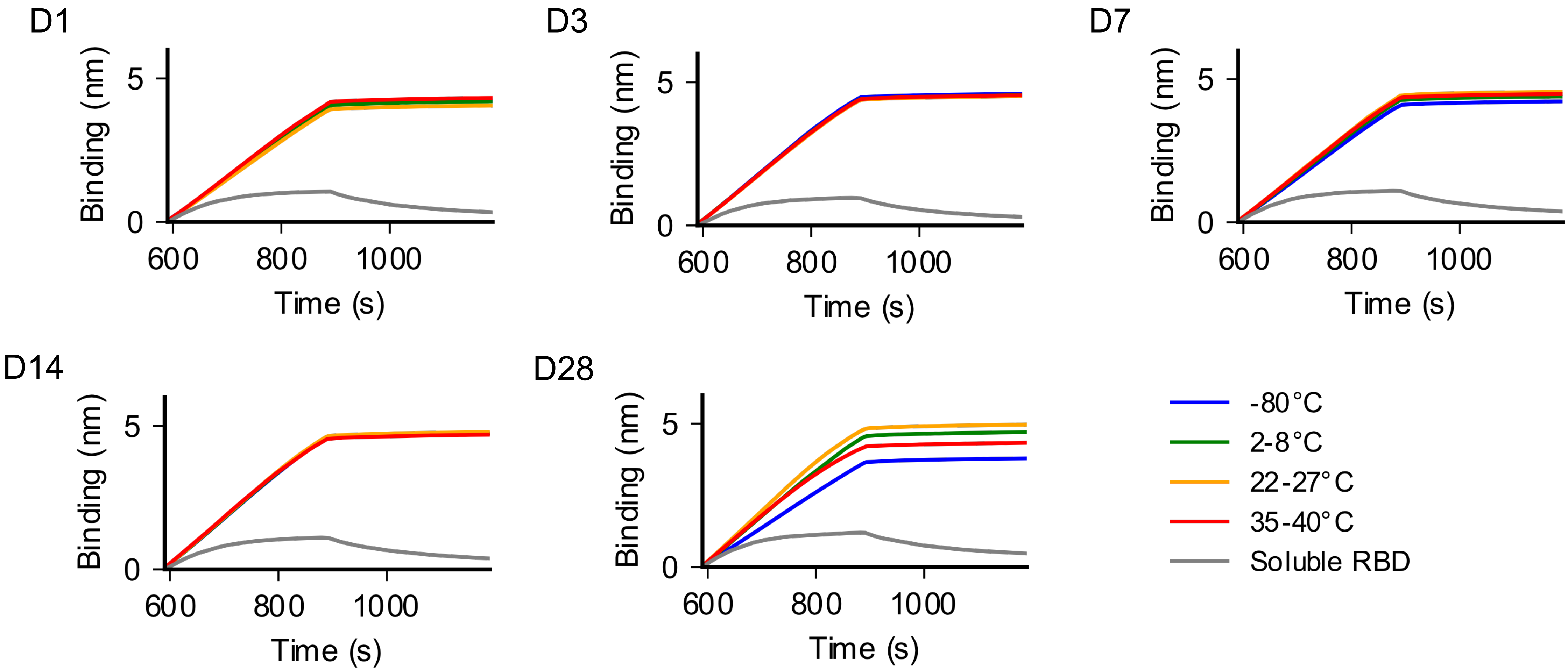

CR3022 IgG binding of antigen incubated at four different temperatures for 28 days (D) was analyzed by bio-layer Interferometry (BLI). Protein A biosensors loaded with CR3022 were incubated with immunogen (association, x = 590–889 s) and then buffer (dissociation, x = 890–1190 s).

nsEM for Rpk4-I53-50 nanoparticle

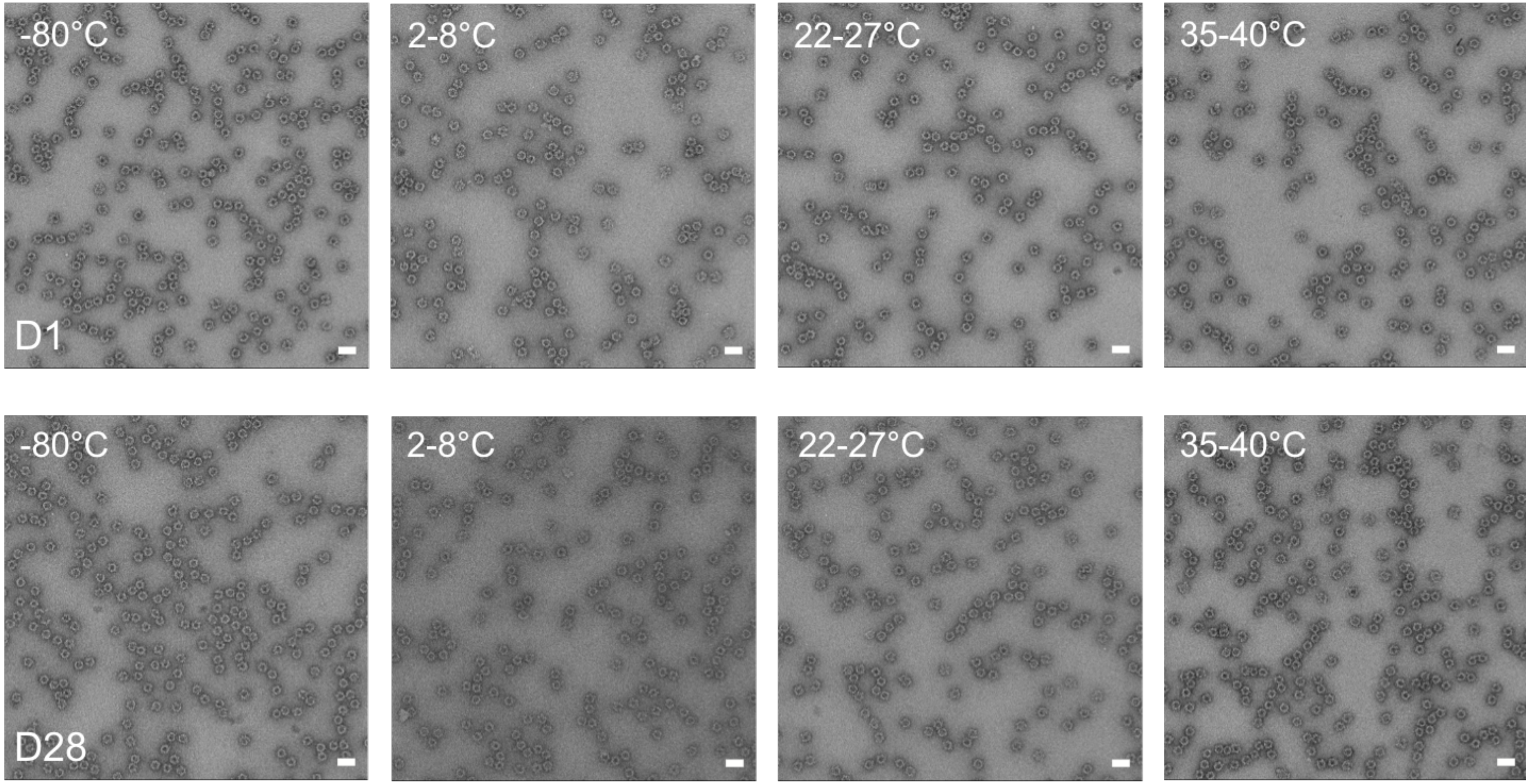

Representative negative stain electron micrographs for each sample at days (D) 1 and 28 following incubation at four temperatures. Scale bar, 50 nm.

Dynamic light scattering for Rpk4-I53-50 nanoparticle

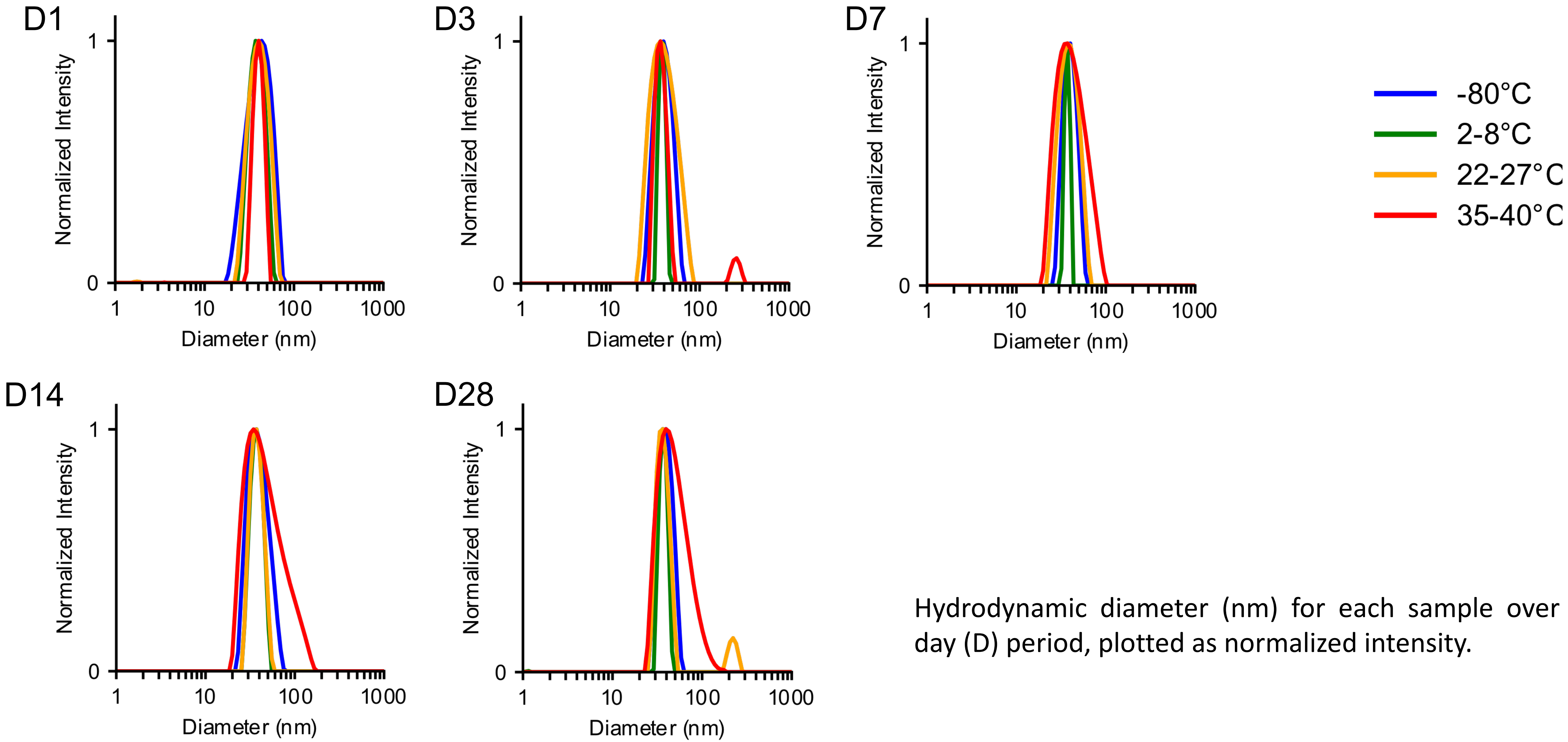

Hydrodynamic diameter (nm) for each sample over a 28 day (D) period, plotted as normalized intensity.

SDS-PAGE for Rpk9-I53-50 nanoparticle

D1

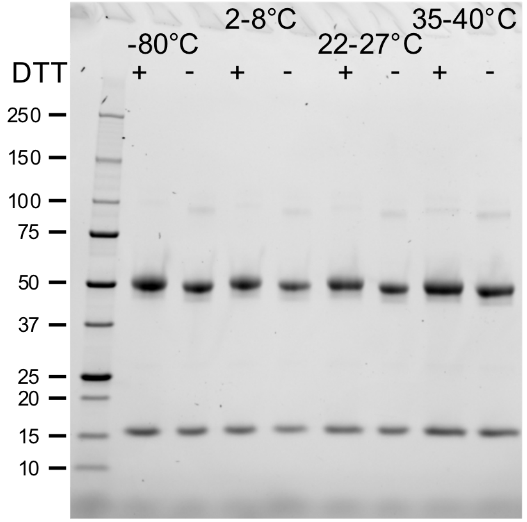

D3

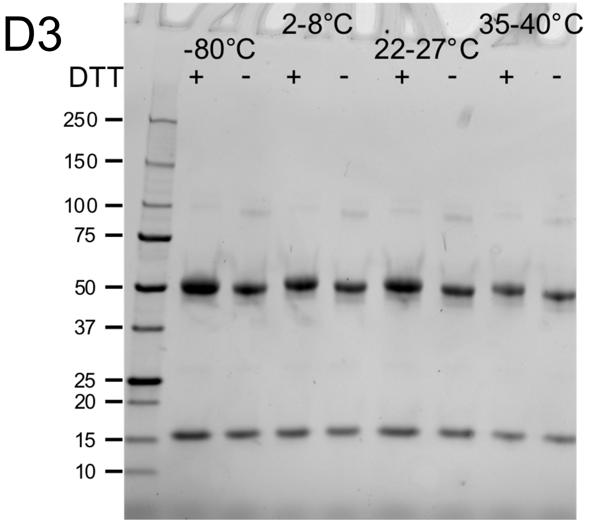

D7

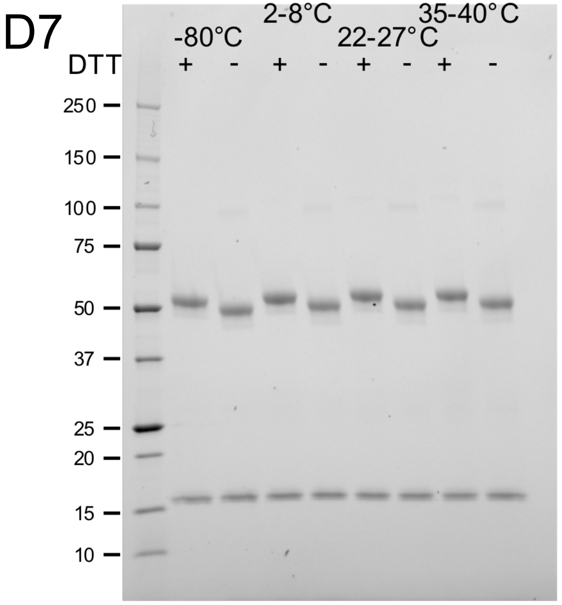

D14

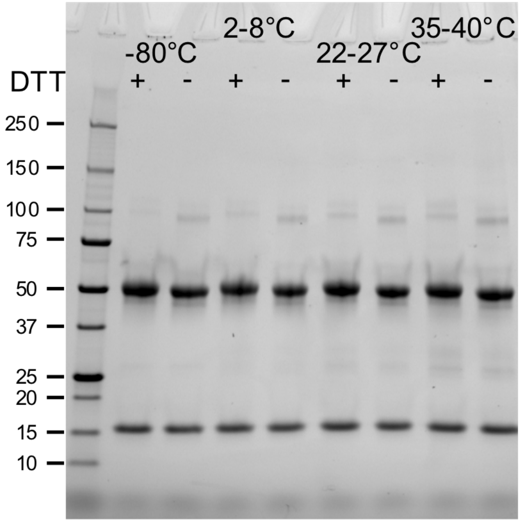

D28

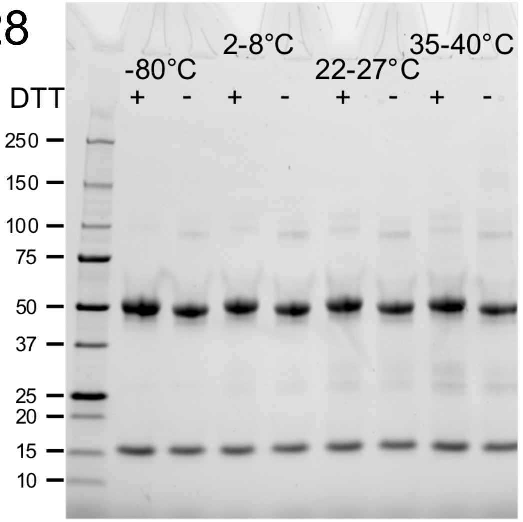

The integrity of samples after incubation at four temperatures over a 28 day (D) study was analyzed by SDS-PAGE. Molecular weights of the standard are noted in kDa. Each sample was analyzed +/- reducing agent (DTT).

hACE2-Fc binding for Rpk9-I53-50 nanoparticle

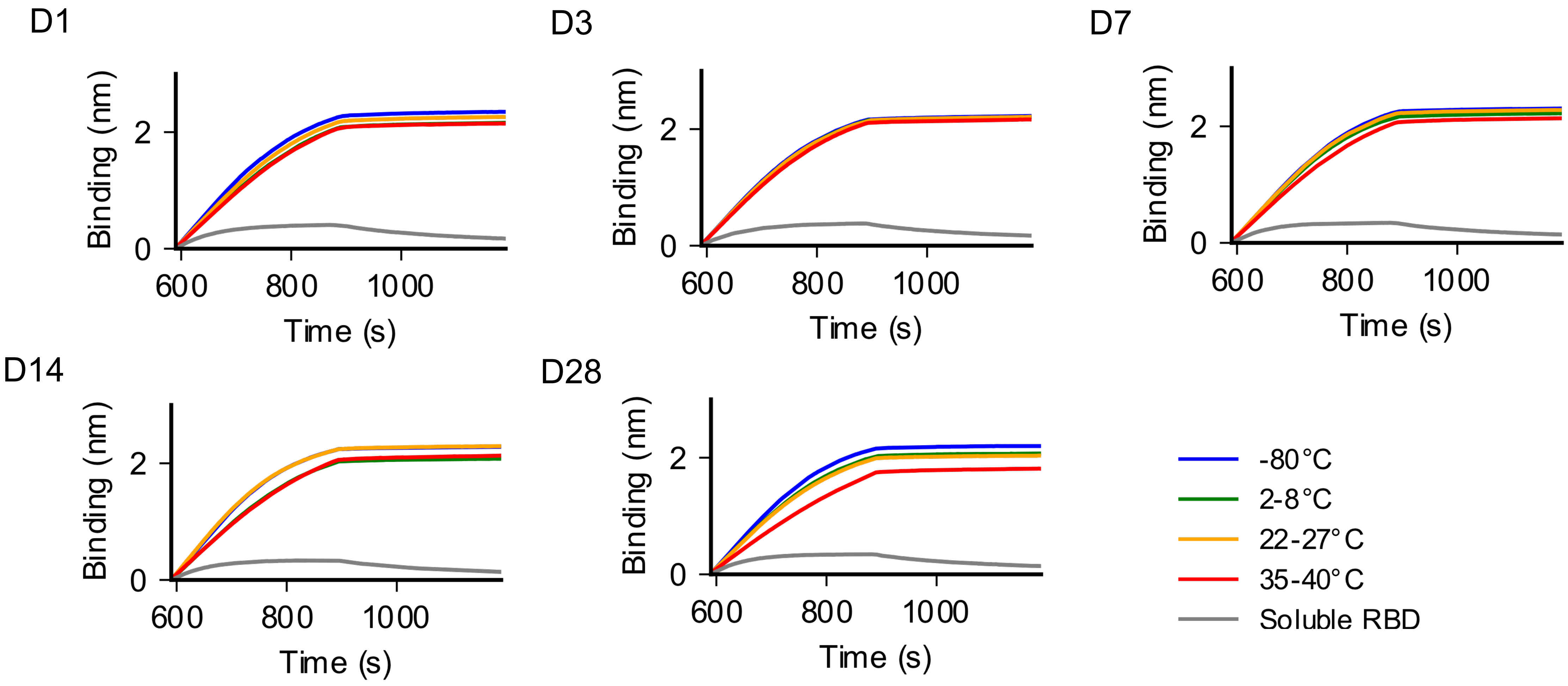

hACE2-Fc binding of antigen incubated at four different temperatures for 28 days (D) was analyzed by bio-layer Interferometry (BLI). Protein A biosensors loaded with hACE2-Fc were incubated with immunogen (association, x = 590–889 s) and then buffer (dissociation, x = 890–1190 s).

CR3022 binding for Rpk9-I53-50 nanoparticle

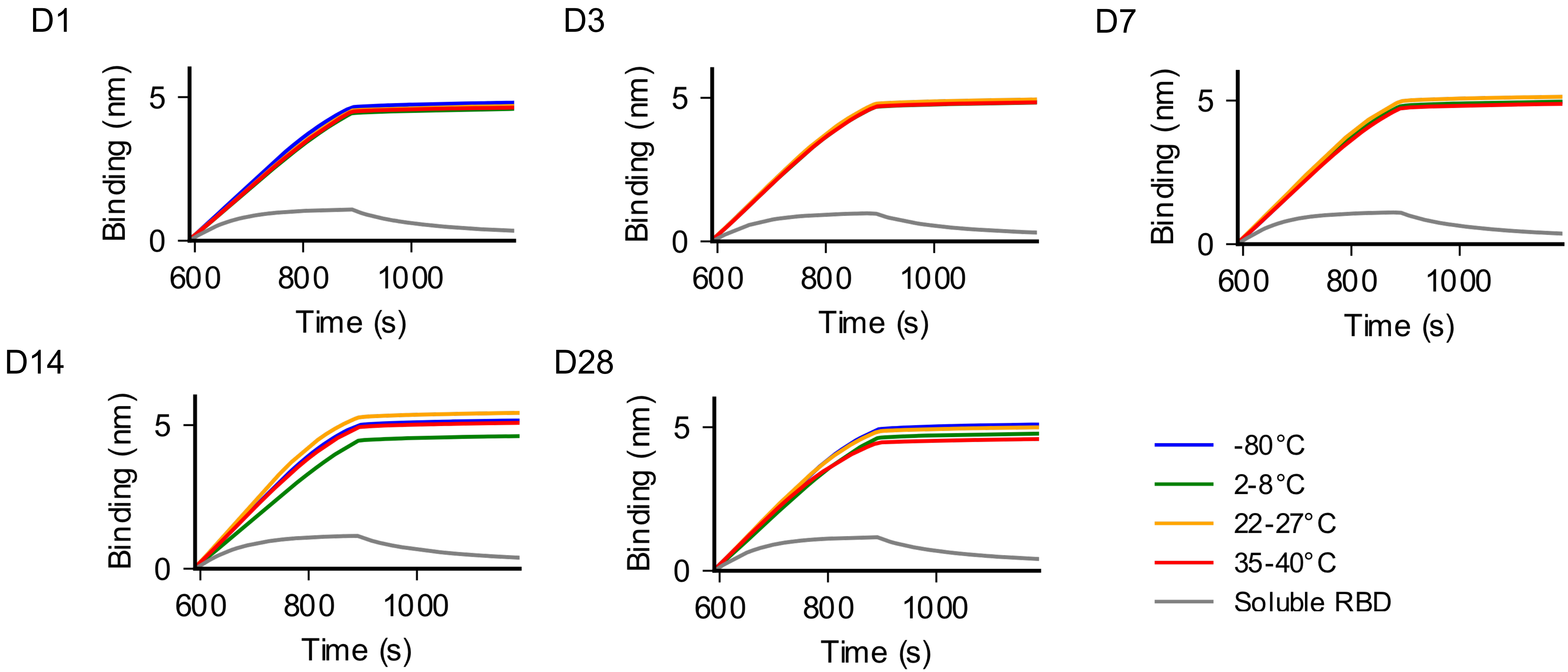

CR3022 IgG binding of antigen incubated at four different temperatures for 28 days (D) was analyzed by bio-layer Interferometry (BLI). Protein A biosensors loaded with CR3022 were incubated with immunogen (association, x = 590–889 s) and then buffer (dissociation, x = 890–1190 s).

nsEM for Rpk9-I53-50 nanoparticle

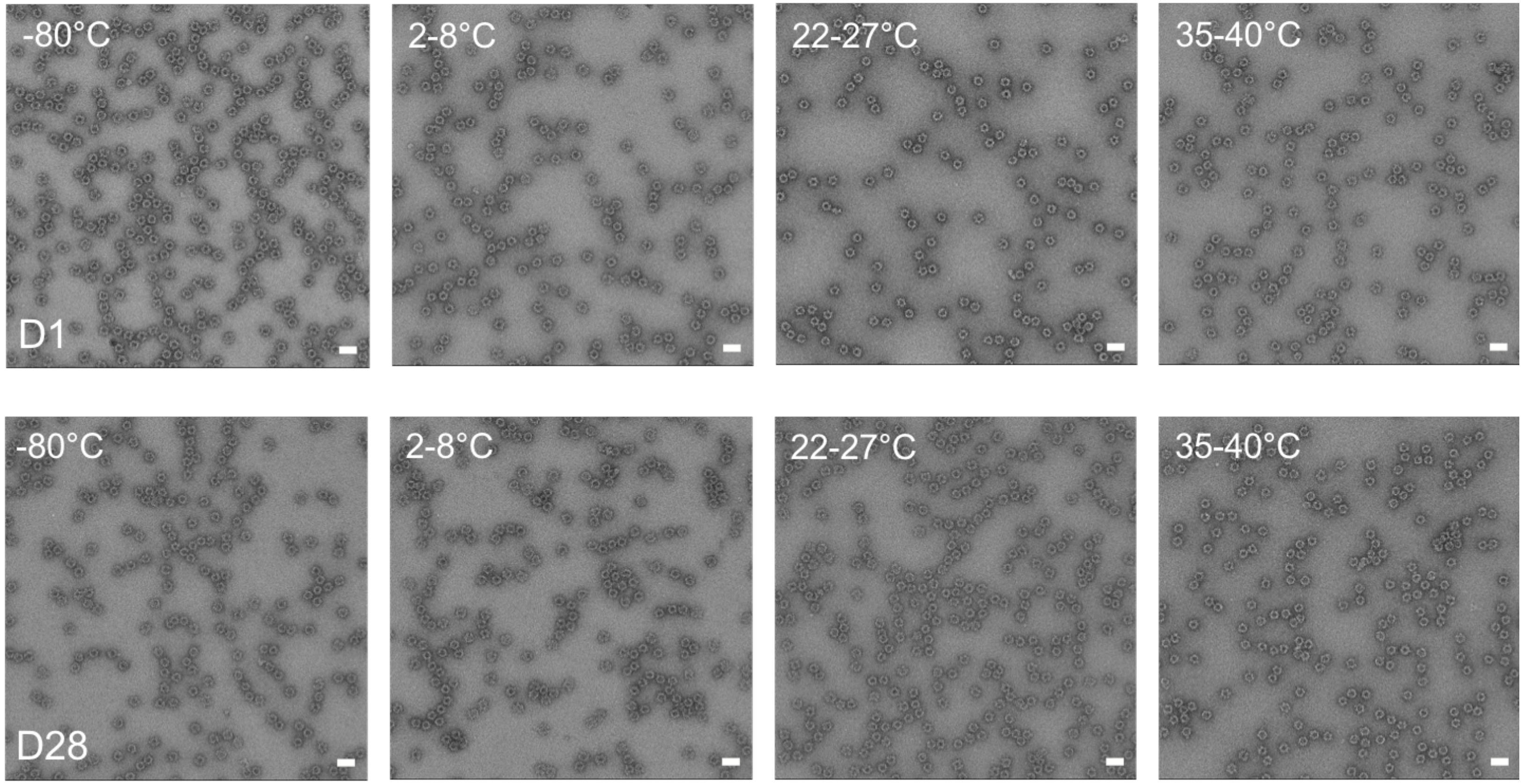

Representative negative stain electron micrographs for each sample at days (D) 1 and 28 following incubation at four temperatures. Scale bar, 50 nm.

Dynamic light scattering for Rpk9-I53-50 nanoparticle

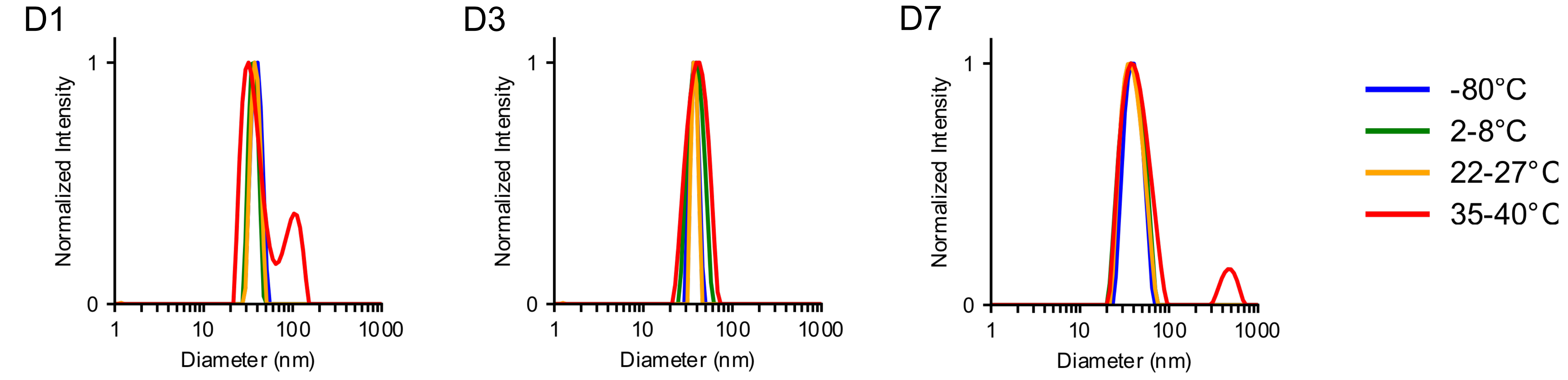

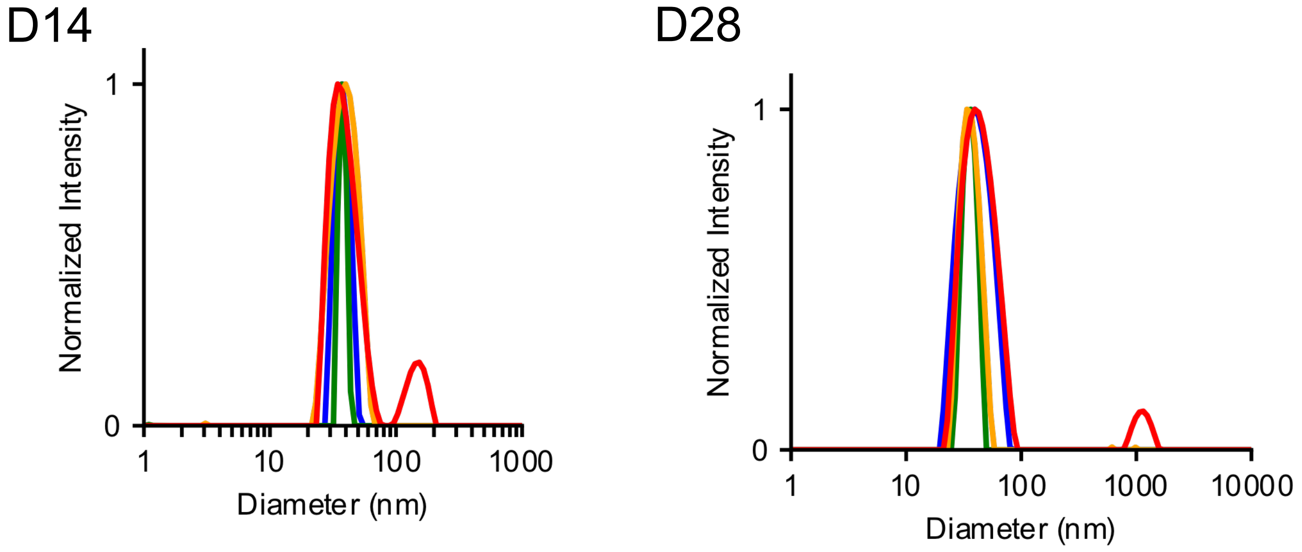

Hydrodynamic diameter (nm) for each sample over a 28 day (D) period, plotted as normalized intensity.
